# Genome-wide transcriptional analysis suggests hydrogenase- and nitrogenase-mediated hydrogen production in *Clostridium butyricum* CWBI 1009

**DOI:** 10.1186/s13068-015-0203-5

**Published:** 2015-02-22

**Authors:** Magdalena Calusinska, Christopher Hamilton, Pieter Monsieurs, Gregory Mathy, Natalie Leys, Fabrice Franck, Bernard Joris, Philippe Thonart, Serge Hiligsmann, Annick Wilmotte

**Affiliations:** Centre for Protein Engineering, Bacterial Physiology and Genetics, University of Liège, Allée de la Chimie 3, B-4000 Liège, Belgium; Walloon Centre of Industrial Biology, University of Liège, Boulevard du Rectorat 29, B-4000 Liège, Belgium; Microbiology Unit, Expertise Group for Molecular and Cellular Biology, Institute for Environment, Health and Safety, Belgian Nuclear Research Centre (SCK-CEN), Boeretang 200, B-2400 Mol, Belgium; Bioenergetics Laboratory, University of Liège, Boulevard du Rectorat 27, B-4000 Liège, Belgium; Environmental Research and Innovation Department, Luxembourg Institute of Science and Technology, Rue du Brill 41, L-4422 Belvaux, Luxembourg

**Keywords:** Dark fermentation, *Clostridium butyricum*, [FeFe] hydrogenase, Nitrogenase, RNA-seq, 2D-DIGE

## Abstract

**Background:**

Molecular hydrogen, given its pollution-free combustion, has great potential to replace fossil fuels in future transportation and energy production. However, current industrial hydrogen production processes, such as steam reforming of methane, contribute significantly to the greenhouse effect. Therefore alternative methods, in particular the use of fermentative microorganisms, have attracted scientific interest in recent years. However the low overall yield obtained is a major challenge in biological H_2_ production. Thus, a thorough and detailed understanding of the relationships between genome content, gene expression patterns, pathway utilisation and metabolite synthesis is required to optimise the yield of biohydrogen production pathways.

**Results:**

In this study transcriptomic and proteomic analyses of the hydrogen-producing bacterium *Clostridium butyricum* CWBI 1009 were carried out to provide a biomolecular overview of the changes that occur when the metabolism shifts to H_2_ production. The growth, H_2-_production, and glucose-fermentation profiles were monitored in 20 L batch bioreactors under unregulated-pH and fixed-pH conditions (pH 7.3 and 5.2). Conspicuous differences were observed in the bioreactor performances and cellular metabolisms for all the tested metabolites, and they were pH dependent. During unregulated-pH glucose fermentation increased H_2_ production was associated with concurrent strong up-regulation of the nitrogenase coding genes. However, no such concurrent up-regulation of the [FeFe] hydrogenase genes was observed. During the fixed pH 5.2 fermentation, by contrast, the expression levels for the [FeFe] hydrogenase coding genes were higher than during the unregulated-pH fermentation, while the nitrogenase transcripts were less abundant. The overall results suggest, for the first time, that environmental factors may determine whether H_2_ production in *C. butyricum* CWBI 1009 is mediated by the hydrogenases and/or the nitrogenase.

**Conclusions:**

This work, contributing to the field of dark fermentative hydrogen production, provides a multidisciplinary approach for the investigation of the processes involved in the molecular H_2_ metabolism of clostridia. In addition, it lays the groundwork for further optimisation of biohydrogen production pathways based on genetic engineering techniques.

**Electronic supplementary material:**

The online version of this article (doi:10.1186/s13068-015-0203-5) contains supplementary material, which is available to authorized users.

## Background

Molecular hydrogen has great potential as a clean energy vector given its pollution-free combustion and the ease with which it can be converted into electricity via fuel cells. However, current industrial hydrogen production processes, such as steam reforming of natural gas, release large quantities of CO_2_ and thereby contribute substantially to the greenhouse effect [1]. Consequently, scientific interest in recent years has focused on alternative methods of hydrogen production, in particular on the use of photosynthetic and fermentative microorganisms for CO_2_-neutral H_2_ production from renewable energy sources, such as solar energy and biomass [2]. Nevertheless, a major challenge when using microorganisms for H_2_ production is the low yield generally obtained, with typical mesophilic fermentation of carbohydrates supplying only 10 to 20% of the H_2_ potentially available in the substrate. Consequently, research in the field of metabolic engineering has investigated different approaches with a view to optimising the yield of well-characterised biohydrogen production pathways [3]. However, such strategies require a thorough and detailed understanding of the relationships between genome content, gene expression patterns, pathway utilisation and metabolite synthesis.

In dark anaerobic fermentation microorganisms break down carbohydrate-rich substrates into organic acids and alcohols while releasing H_2_. Strict anaerobes such as clostridia have been the most widely studied among the various anaerobic and facultative anaerobic bacteria capable of fermentative hydrogen production [4]. H_2_ production in living organisms is always dependent on the presence of H_2_-producing enzymes such as hydrogenases and nitrogenases. [FeFe] hydrogenases, which are especially abundant in clostridia, are well recognised as the main H_2_-producing enzymes in this genus [5,6]. By contrast, nitrogenase-mediated hydrogen production has never been proposed for clostridia, even though this is known to be an intrinsic metabolic property of many cyanobacteria and photosynthetic bacteria [2]. Interestingly, in 1960 Carnahan showed that the free-living soil microorganism *Clostridium pasteurianum* is an efficient N_2_ fixator [7]. Later *nif* operons were described in *C. acetobutylicum* and *C. beijerinckii* [8], but the contribution of nitrogenase to overall H_2_ production in clostridia has not yet been reported in the literature.

*Clostridium butyricum* CWBI 1009, a strain recently isolated from an anaerobic sludge, was previously shown to ferment different carbon substrates at acidic pH to H_2_ and CO_2_ with formate, butyrate and acetate as the main end-products [9,10]. It possesses four different [FeFe] hydrogenases, three of which are monomeric belonging to clusters A2, B2 and B3, and one which is a trimeric enzyme representing cluster A8 [6,11]. Despite the fact that optimum growth for this bacterium occurs at pH 7.3, it only starts to produce H_2_ when the pH declines due to the natural acidification of the medium as fermentation proceeds. The optimal pH value to produce H_2_ under fixed-pH culture conditions was found to be 5.2 and has been discussed in previous work [9]. Although many studies have described the fermentative activity of *C. butyricum* associated with H_2_ production [12,13], a comprehensive analysis of the fermentative pathways at the genomic and proteomic levels has not yet been reported. Relative gene expression profiles, together with the associated proteomic and metabolite data, can now be used to provide the visibility needed for well-targeted metabolic engineering. Moreover, a better understanding of the shifts in gene and protein expression, which occur in response to pH changes and during the growth phase, should facilitate the optimisation of bioreactor performance.

Therefore, in this study three parallel approaches were used to investigate the changes at the molecular level associated with pH-dependent hydrogen production in *C. butyricum* CWBI 1009, namely metabolite analysis, transcriptomics and proteomics. The effect of the naturally decreasing pH was studied with glucose in 20-L batch bioreactors. Additionally, the effect of fixed-pH fermentations was evaluated to provide comparative data under optimal pH conditions for cellular growth (pH 7.3) and H_2_ production (pH 5.2), respectively. The genome of *C. butyricum* CWBI 1009, which was unknown until now, was also sequenced to provide better mapping of the RNA-sequencing (RNA-seq) reads during natural acidification of the medium. In addition the expression levels of the H_2_-producing enzymes, namely the [FeFe] hydrogenases and the nitrogenase, were determined under different pH conditions.

## Results and discussion

### Experimental design

**Figure 1:**
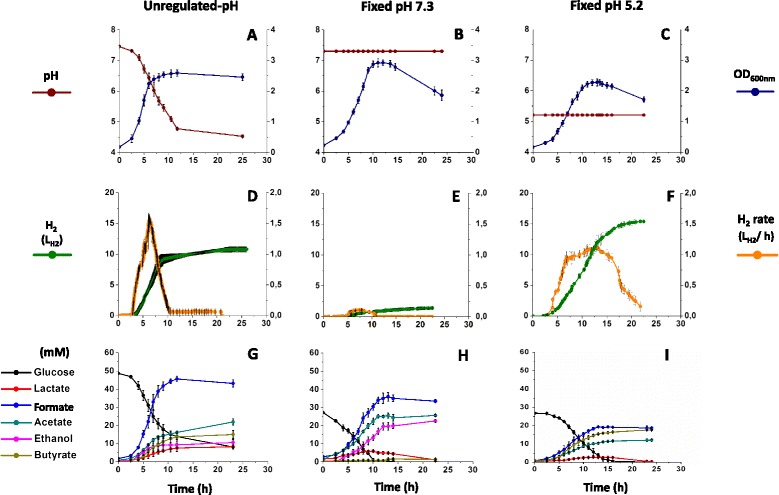
**Characteristics of the different glucose fermentations for** ***Clostridium butyricum*** **CWBI 1009 performed under unregulated-pH and at fixed pH 7.3 and 5.2. (A, B, C)** Growth curve (OD) and pH. **(D, E, F)** Hydrogen production rate (L/h) and cumulative hydrogen production (L) profiles. **(G, H, I)** Glucose utilisation and profiles of soluble metabolites (mM). The error bars refer to the three independent replicate experiments. Fermentations were performed in 20-L batch bioreactors with glucose as a substrate (10 g/L for unregulated-pH fermentations and 5 g/L for fixed-pH fermentations).

Three 20-L glucose fermentations were carried out under unregulated-pH conditions (Figure 1, A, D and G), allowing characterisation of the impact of naturally decreasing pH on fermentative H_2_ production by *C. butyricum* CWBI 1009. Additional glucose fermentations (three replicates for each condition) performed at two fixed pH values, namely 7.3 (Figure 1, B, E and H) and 5.2 (Figure 1, C, F and I), were carried out to provide comparative data for the interpretation of the results obtained during the unregulated-pH fermentations. These fixed pH values of 7.3 and 5.2 were previously determined to be optimal for *C. butyricum* CWBI 1009 growth and H_2_ production, respectively [9]. The fermentation results presented below for the triplicated experiments were characterised by standard deviations ranging from 5 to 10% of the absolute value. Therefore, the reproducibility of the results may be considered as sufficient and typical for these kinds of experiments in 20-L bioreactors.

For all the above fermentations, analyses were carried out to determine whether the H_2_ production and the cellular metabolism for all the tested metabolites varied and, if so, to what extent these variations were pH dependent. The genome-wide transcriptional response of *C. butyricum* CWBI 1009 to naturally decreasing pH (associated with increasing H_2_ production) was characterised during glucose fermentation without pH regulation. The gene expression levels of the H_2_-producing enzymes, namely the [FeFe] hydrogenases and the nitrogenase, were then examined, and the data were related to the H_2_-production profiles under the different conditions studied. Finally the proteomic response of *C. butyricum* CWBI 1009 to declining pH was analysed.

### H_2_ and metabolite production under unregulated-pH and fixed pH conditions

The effect of naturally decreasing pH on the growth, H_2_-production and fermentation profiles of *C. butyricum* CWBI 1009 cultivated with glucose (10 g/L) was evaluated in a 20-L batch anaerobic bioreactor maintained at 30°C and N_2_ atmosphere. The culture was monitored during the 25 h of fermentation by which time the pH had dropped naturally to about 4.5 (Figure 1A). Directly after the lag phase there was a rapid consumption of glucose and an increase in growth. In total 17.16 ± 0.84 L of biogas were produced. Given that the average H_2_ content of the biogas was 63 ± 4%, it was calculated that 10.80 ± 0.44 L of H_2_ were produced (Additional file 1: Table S1). The highest H_2_ flow rate, 1.56 ± 0.15 L H_2_/h, was recorded after 6 to 7 h of fermentation (Figure 1D) by which time the pH of the medium had dropped to around 6.3. The highest calculated H_2_ yield was 1.78 ± 0.11 mol H_2_/mol glucose. The primary soluble metabolites at the end of fermentation were formate, acetate and butyrate, followed by ethanol and lactate (Figure 1G).

To highlight the impact of pH on the gene expression for the H_2_-producing enzymes, that is, the hydrogenases and the nitrogenase, the comparative results for H_2_ and metabolite production with glucose (5 g/L), using the same bioreactor, are briefly presented for two different fixed-pH conditions: pH 7.3 and 5.2 ± 0.15. At fixed pH 7.3 a lag phase of 3 h was observed (Figure 1B), compared to a lag phase of 4.5 h at fixed pH 5.2 (Figure 1C). Exponential growth followed the lag phase under both pH conditions. At pH 7.3 rapid consumption of glucose was observed (Figure 1H), and the glucose uptake rate peaked at 0.96 ± 0.08 g glucose/h. Biogas production started after 5.5 h of fermentation, yielding a total of 2.90 ± 0.23 L of biogas after 15 h of fermentation (Additional file 1: Table S2). At pH 5.2, the glucose uptake rate was much lower (0.44 ± 0.03 g glucose/h) and biogas production had already started after 3.5 h of fermentation. After 20 h of fermentation a total of 23.32 ± 1.01 L of biogas was produced. At pH 7.3 the H_2_ production rate and yield peaked at 0.21 L ± 0.03 H_2_/h (Figure 1E) and 0.23 ± 0.02 mol H_2_/mol glucose, respectively. By contrast, at pH 5.2 the corresponding H_2_ production rate and yield were much higher; 1.11 L ± 0.06 H_2_/h (Figure 1F) and 1.95 ± 0.09 mol H_2_/mol glucose. The cellular metabolisms for all the tested metabolites varied between the unregulated-pH fermentations and those when the pH was fixed at 5.2 and 7.3. Moreover, the lactate produced during the early stages of fermentation was later consumed, which was not the case with the fermentations carried out under unregulated-pH conditions (Figure 1H and I). The ability of clostridia to reconsume lactate produced during the early stages of fermentation indicates the existence of novel metabolic pathways, an observation that has already been discussed in the literature [10].

### Transcriptional response of *C. butyricum* CWBI 1009 to decreasing pH

**Figure 2:**
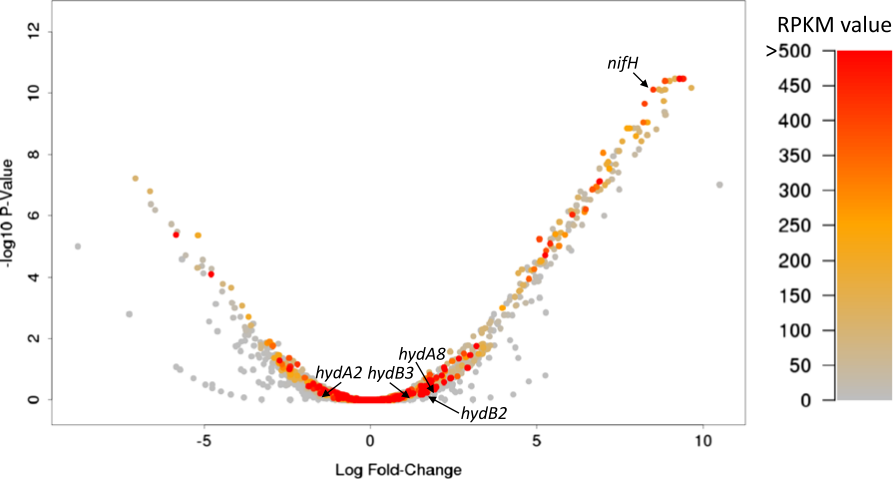
**Volcano plot distribution of** ***Clostridium butyricum*** **CWBI 1009 mRNA transcript levels (RNA-seq) during unregulated-pH glucose fermentation.** The colour code corresponds to the expression level and is presented as an RPKM value for pH 6.3. Dots corresponding to the *hydA2, hydA8, hydB2, hydB3* and *nifH* genes are indicated.

**Figure 3:**
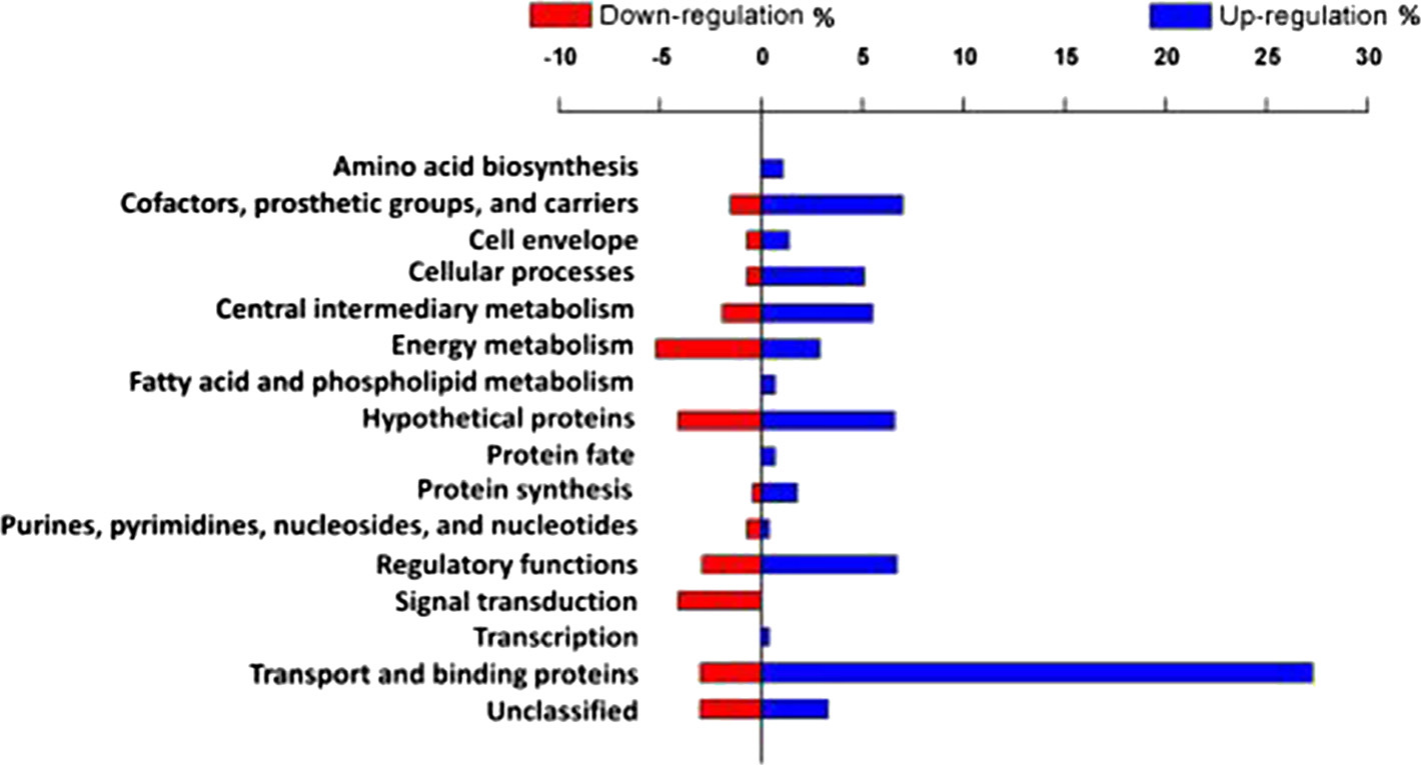
**Differentially regulated pathways of** ***Clostridium butyricum*** **CWBI 1009 during unregulated-pH glucose fermentation based on the RNA-seq data (pH 6.3 versus pH 7.3).**

As an initial step towards understanding how the bacterium responds to naturally decreasing pH, which is associated with increasing H_2_ production, the transcriptional response of *C. butyricum* CWBI 1009 was analysed during unregulated-pH glucose fermentation. The genome of *C. butyricum* CWBI 1009 was also sequenced to provide better mapping of the RNA-seq reads. The RNA-seq data were obtained from rRNA-depleted mRNA samples isolated from two independent reactor cultures (biological replicates). RNA-seq data were acquired from samples taken at pH 7.3 (early exponential growth phase: control sample) and at pH 6.3 (late exponential growth phase: test samples), and are shown in Additional file 2: Table S4 and Additional file 3: Table S5. The selection of these values was based on the fact that pH 7.3 and 6.3 corresponded respectively to the minimum and maximum H_2_ production phases of the three fermentations without pH regulation. Additionally, the gene expression profiles during the stationary phase, corresponding in this experiment to pH 5.2, were analysed and are shown in Additional file 2: Table S4. The reproducibility of the transcriptomic data between the two biological replicates was high with an R^2^ ranging from 0.873 for the control sample (pH 7.3) to 0.78 for the samples taken at pH 6.3 (Additional file 1: Figure S1). For each sample both the reads mapping to rRNA sequences and those not mapping uniquely to the genome of *C. butyricum* CWBI 1009 were omitted from further analysis (Additional file 1: Table S3). The RNA-seq expression data have been presented in two ways. Firstly, the total number of reads for each coding DNA sequence (CDS) was calculated and converted to reads per kilobase per million mapped reads (RPKM numbers), and secondly the genes that were differentially regulated between the stages corresponding to pH 6.3 and 7.3 were identified (Figures 2 and 3).

Calculation of the RPKM enabled a comparison between the relative mRNA abundances of different genes for a given condition and also between the relative abundances of a specific gene under the different pH conditions. Based on the RPKM numbers, the genes coding for the glycolytic enzymes and the auxiliary proteins involved in the core metabolic reactions (for example, ferredoxin, NAD-dependent glyceraldehyde-3-phosphate dehydrogenase, pyruvate formate-lyase, acetaldehyde dehydrogenase, pyruvate kinase or flavodoxin) were among the most highly expressed, and their expression did not vary significantly throughout the fermentation (Additional file 2: Table S4). Unexpectedly, multiple genes encoding different subunits of the nitrogenase (for example, *nifN*, *nifH*, *nifD*, *nifS*) and the urease (α, β and γ subunits) were also very highly expressed at pH 6.3 (RPKM ≥ 500). To our surprise the mRNA moieties of the [FeFe] hydrogenases were among the least abundant transcriptional units, which contrasts strongly with the increased H_2_ production associated with this fermentation stage (Figure 2).

RNA-seq data were also used to identify the genes that were significantly up- or down-regulated under the different pH conditions studied. The relative expression levels were presented as a fold change (log_2_) between the control sample (pH 7.3) and the test sample (pH 6.3). In total more than 290 genes were found to be differentially expressed, with 72% being up-regulated and only 28% down-regulated at pH 6.3 (Figure 3, Additional file 3: Table S5). Many of these genes were located in close proximity on the chromosome, and are therefore likely to represent polycistronic operons encoding proteins with similar functions. The differentially regulated genes were automatically assigned functional annotations using the Clusters of Orthologous Groups (COG) database; these annotations were then corrected based on the Pathema-*Clostridium* assigned categories (http://pathema.jcvi.org).

Over 30% of the annotated and differentially regulated transcripts were associated with transport proteins (28% of which were up-regulated), suggesting that transport plays a crucial role in maintaining cell homeostasis at acidic pH. Sequences linked to the biosynthesis of cofactors, prosthetic groups and carriers, as well as the central intermediary metabolism and regulatory function proteins were also found to be differentially regulated (Figure 3). Genes encoding for conserved hypothetical proteins constituted around 10% of all the differentially regulated genes, and a few of them had very high RPKM values at pH 6.3 (RPKM ≥ 1,000, Additional file 2: Table S4), suggesting their importance for cell metabolism. In line with this result, Wang *et al*. [14] reported that many genes encoding for hypothetical proteins accounted for a large fraction of the highly expressed genes during the different stages of batch glucose fermentation by *Clostridium beijerinckii* NCIMB 8052. In addition, the genes coding for the proteins involved in protein folding and stabilisation, such as heat shock proteins, were not differentially expressed, but were nevertheless very highly expressed throughout the fermentation (RPKM ≥ 1,000 for *dnaK*, *groEL* and *groES*). For comparison, genes such as *groESL, hsp90 or dnaK* were previously reported to be induced by acetate and butyrate shock in *C. acetobutylicum* [15] and also by butanol in this bacterium [16] and were confirmed as being important in the general stress response.

#### Is H_2_ production under unregulated-pH conditions nitrogenase-mediated?

**Figure 4:**
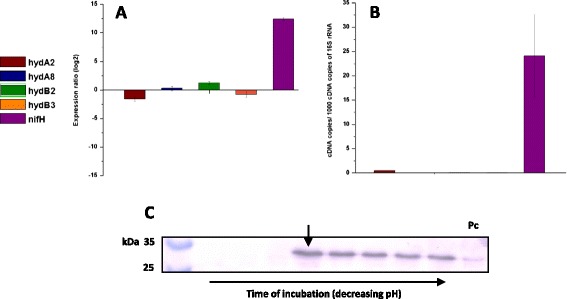
**Relative expression of [FeFe] hydrogenases and** ***nifH*** **genes determined by RT-qPCR of** ***Clostridium butyricum*** **CWBI 1009 during unregulated-pH glucose fermentation.** Western blot analysis for the NifH subunit. (A) Fold change in the expression level of *hydA2, hydA8, hydB2, hydB3, nifH* genes during the unregulated-pH glucose fermentation determined by RT-qPCR. The values correspond to the pH 6.3 (test) sample) versus pH 7.3 (control) sample). **(B)** Basal expression levels of *hydA2, hydA8, hydB2, hydB3, nifH* gene transcripts during the unregulated-pH glucose fermentation. The values correspond to the late exponential growth stage that refers to the peak in H_2_ production at pH 6.3. Expression level is shown as number of cDNA copies per 1,000 cDNA copies of 16S rRNA. **(C)** Western blot analysis of the crude cellular extracts taken during the unregulated-pH glucose fermentation. Time of incubation corresponds to the different growth stages starting from the beginning of the experiment, until the pH dropped to a level of about 4.5. The arrow indicates pH 6.3. Pc: positive control.

The genome of *C. butyricum* CWBI 1009 encodes four [FeFe] hydrogenases. However, the RNA-seq analysis unexpectedly indicated that none of them showed any signs of being up-regulated at pH 6.3, when the H_2_ production rate was maximum (Figure 2, Additional file 3: Table S5). Furthermore, this result was confirmed by RT-qPCR with hydrogenase-specific primers for each gene (Figure 4A and B, Additional file 1: Figure S2).

Surprisingly, the genes that were among the most strongly induced at pH 6.3 included the genes encoding nitrogenase and other proteins related to N_2_ fixation, ammonium transport and molybdenum transport (Additional file 3: Table S5). The physiological electron donors for nitrogenases, ferredoxins (RPKM > 8,800) and flavodoxins (RPKM > 4,700) were also very highly abundant at pH 6.3 (Additional file 2: Table S4). In addition various nitrogen regulatory P-II proteins were significantly up-regulated at pH 6.3 and accounted for 30% of the regulatory factors that were differentially regulated between the two pH conditions studied (Additional file 4: Table S8).

Nitrogenase-mediated H_2_ production is a property of several microorganisms including cyanobacteria and photosynthetic bacteria [2], and has recently been revealed in marine *Enterobacteriaceae*, for example, *Pantoea agglomerans* [17]. However, it has never before been proposed for clostridia. Therefore, to validate the up-regulation of the nitrogenase genes, an RT-qPCR analysis was carried out for the *nifH* gene, which encodes a nitrogenase reductase subunit. The results showed a significant up-regulation of this gene at pH 6.3 and were in agreement with the RNA-seq results (Figure 4A, Additional file 1: Figure S2). Additionally, by using anti-NifH antibodies, the presence of the nitrogenase H subunit in crude cellular extracts was confirmed by Western blot analysis (Figure 4C). Since the H_2_ production rate peaked at 1.56 ± 0.15 L/h at pH 6.3 and was below 0.21 ± 0.03 L/h at pH 7.3 (Figure 1D), the very strong up-regulation of the nitrogenase coding genes at the lower pH, combined with the concurrent absence of up-regulation of the [FeFe] hydrogenase genes, led us to conclude that the H_2_ production in *C. butyricum* CWBI 1009 during glucose fermentation with unregulated- pH may be nitrogenase-mediated.

Interestingly, though nitrogenase is known to produce H_2_ as a by-product of N_2_ fixation, already in the early 1980s it was reported that the enzyme may act as an ATP-powered hydrogenase and produce only H_2_ in the absence of N_2_ [18]. Therefore, to check if this was the case with clostridia, *C. butyricum* CWBI 1009 was cultured in an N_2_-free atmosphere with unregulated- pH, using argon instead of nitrogen to initiate the anaerobic conditions in the bioreactor. The preliminary results demonstrated that, in contrast to the fermentation with unregulated- pH under N_2_, under an Ar atmosphere there was an induction of three [FeFe] hydrogenase genes (*hydA8, hydB2* and *hydB3)* at low pH values (results discussed in Additional file 6). A better understanding of the differences between the mechanisms leading to H_2_ production under these two conditions would nevertheless require a more detailed analysis of the Ar sample at the transcriptomic and proteomic levels. Additionally, as a follow-up to the present work, a study of the physiological activity of the [FeFe] hydrogenases and the nitrogenase could be carried out to evaluate the exact contribution of each enzyme to the overall H_2_ production under different experimental conditions.

#### The physiological function of hydrogen and ammonia-generating enzymes under unregulated-pH conditions may be to maintain the pH homeostasis of the cell

**Figure 5:**
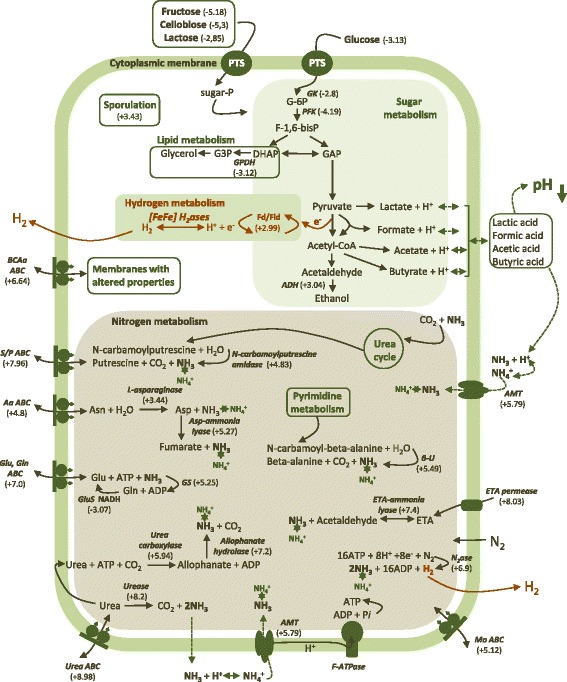
**Metabolic pathways for** ***Clostridium butyricum*** **CWBI 1009 affected by decreasing pH during the unregulated-pH glucose fermentation.** The numbers are the calculated averages of the individual calculated expression ratios when several subunits encoding the same enzyme were detected to be differentially expressed by RNA-seq. Pathways directly related to H_2_ production are indicated in red. The dashed lines refer to pathways involved in pH regulation. *AaABC*: amino acid ABC transporter; *ADH*: alcohol dehydrogenase; *AMT*: ammonium transporter; *β-U*: Beta-ureidopropionase; *BCAa ABC*: branched-chain amino acid ABC transporter; *ETA: ethanolamine permease*; *Fd/Fld:* ferredoxin, flavodoxin; *GK:* glucokinase; *Glu/Gln ABC:* glutamate/glutamine ABC transporter; *GluS*: NADH-glutamate synthase; *GS:* glutamine synthetase; *[FeFe] H*_*2*_ *ase:* [FeFe] hydrogenase; *MoABC:* molybdenum ABC transporter; *N*_*2*_ *ase:* nitrogenase; *PFK:* 1-phosphofructokinase; *S/P ABC:* spermidine/putrescine ABC transporter; *Urea ABC:* urea ABC transporter.

N_2_ fixation (reduction of N_2_ to two ammonia molecules) is a highly energy-intensive process, consuming at least 16 ATP molecules per molecule of nitrogen fixed. It has been shown that while ammonium nitrogen can repress the formation of nitrogenase in different species [19], amino acid nitrogen can actually stimulate it (the main N source in this study was the amino acids contained in casein peptone and yeast extract) [20]. The release of molecular H_2,_ a by-product of N_2_ fixation, enables the disposal of excess protons, thereby preventing acidification of the cytoplasm. Clearly proton disposal is the main physiological function of [FeFe] hydrogenases, but in this case nitrogenase activity may provide an additional buffering molecule, namely ammonia (Figure 5).

Cytoplasmic pH buffering is one of the strategies employed by many microorganisms to maintain pH homeostasis, involving the production of various different buffer molecules such as amino acids, ammonia and polyamines, [21]. With declining external pH (the pK for ammonia is 9.25), the ammonia produced may buffer the cytoplasm via the H^+^ + NH_3_ ↔ NH_4_^+^ reaction. Another enzyme for which various subunits were up-regulated sixfold to ninefold (log_2_ scale) was urease. It is a nickel-containing enzyme which catalyses the hydrolysis of urea to ammonia and carbamic acid, with the latter spontaneously hydrolysing to carbonic acid and an additional ammonia molecule [22]. Most bacteria use the products of this reaction for anabolic processes, but at low pH these moieties may be used for cytoplasmic buffering. Additionally, the up-regulation of urea carboxylase and allophanate hydrolase at pH 6.3 may indicate the existence in *C. butyricum* CWBI 1009 of another pathway for urea breakdown that also leads to the formation of ammonia and carbon dioxide. Moreover, at pH 6.3 several additional pathways that could contribute to cytoplasmic pH buffering via ammonia synthesis (for example, L-asparaginase, ethanolamine ammonia-lyase, beta-ureidopropionase, aspartate ammonia-lyase and N-carbamoylputrescine amidase) were also significantly up-regulated (Figure 5, Additional file 4: Table S6).

#### Cellular response to decreasing pH involves differential regulation of ABC transporters and other transport proteins

Molecular pumps, which are unidirectional efflux systems that actively expel various chemical substances or ions from the cytoplasm to the extracellular space, have been shown to play a role in acid tolerance in yeast [23]. ABC transporters are membrane-bound molecular pumps that utilise ATP hydrolysis energy to translocate a large variety of solutes across cellular membranes. In our study numerous genes related to the various ABC transporters were up-regulated following the progressive drop in pH, that is, ABC transporters for spermidine, putrescine, arginine, glutamine, branched-chain amino acids, molybdate and other metal ions (Figure 5, Additional file 4: Table S7). Due to their positive charges, polyamines, including spermidine and putrescine, bind to macromolecules such as DNA, RNA and proteins, exerting a protective effect. In bacteria they have been shown to be involved in stress responses, particularly acid tolerance [24]. They are also involved in various different processes, including regulation of gene expression, cell proliferation, cell signaling and membrane stabilisation [25]. Other more frequently transported compounds were the branched-chain amino acids which are involved in the biosynthesis of membranes with altered properties (fluidity). Their increased transport and biosynthesis in *C. acetobutylicum* in response to butyrate and butanol stresses have been previously described [15].

#### Expression of sporulation genes is strongly induced by decreasing pH

During the late exponential growth phase, when the pH of the medium decreased to 6.3, several genes associated with sporulation were significantly induced, constituting around 5% of the differentially regulated genes (Additional file 4: Table S9). Furthermore, the RNA polymerase sporulation-specific sigma factor, SigE, that was reported to be responsible for the expression of stage II sporulation-specific genes in *Bacillus* [26], was also up-regulated by a factor of 3.34 (log_2_ scale) at pH 6.3. The initiation of endospore formation is usually accompanied by reduced chemotaxis and motility, and such a down-regulation was observed here for two genes, the methyl-accepting chemotaxis protein (MCP) signalling domain and a putative methyl-accepting chemotaxis protein. In contrast to our observations, the expression of sporulation genes in some solventogenic clostridia, for example, *C. acetobutylicum*, has been reported as being largely unaffected by a low pH [15]. Instead, to prevent the collapse of the transmembrane pH gradient, some solventogenic bacteria react by solvent production which allows the external pH to increase. Solventogenic *Clostridium beijerinckii* NCIMB 8052 was reported to initiate sporulation concurrently with the onset of solventogenesis [14]. Although *C. butyricum* CWBI 1009 is also capable of producing solvents, mainly ethanol, it does so concomitantly with the production of acids during the exponential growth phase and at a lower concentration (Figure 1G). Therefore, to keep the internal pH close to the optimum during fermentative growth, *C. butyricum* CWBI 1009 employs various different mechanisms for proton disposal as described above. Additionally, the bacterium appears to initiate sporulation even during the early stages of its fermentative growth to prevent cell lysis and death due to the re-uptake, via the membrane, of the fatty acids produced. This typically occurs when the pH decreases below the pKa value (for example, formate pKa 3.77, acetate pKa 4.76, butyrate pKa 4.83) [27].

### Proteomic response to decreasing pH

While mRNAs are mediators of certain biological functions and provide information on transcriptional patterns, most functions are carried out by proteins. Therefore, to gain a better understanding of clostridial hydrogen metabolism, the changes in the relative protein abundance profiles for *C. butyricum* CWBI 1009 were analysed in response to naturally decreasing pH. For this purpose liquid samples were harvested at pH 7.3 (control sample, Figure 1A) and pH 5.2 (test sample) during the unregulated-pH fermentation_._ The experiment was successfully carried out on two separate biological replicates (two independent cultures). The protein abundances were analysed by two-dimensional difference in gel electrophoresis (2D-DIGE) (Additional file 1: Figure S3). Overall 2,750 spots exhibiting differences (>20%) in the normalised spot-volume ratios were detected; 1,423 of these spots increased and 1,327 decreased in size at pH 5.2 versus pH 7.3. More than 500 protein spots varied significantly and were subjected to in-gel tryptic digestion, followed by mass spectrometry fingerprinting of the resulting peptides. The experiment was successfully carried out on two separate biological replicates (two independent cultures). A total of 166 proteins (97 unique and 69 redundant) with significant Mascot probability-based scores were identified and categorised according to their metabolic functions (Additional file 5: Table S10 and S11). Overall 16.5% of the identified proteins were associated with energy metabolism, 10.3% with protein synthesis, 8.2% with signal transduction and 7.2% with amino acid biosynthesis. Though none of the differentially abundant protein spots were identified as [FeFe] hydrogenases or nitrogenase, a differential abundance of these two enzymes between the two pH conditions cannot be excluded since not all of the statistically differentially abundant proteins were identified after matrix-assisted laser desorption/ionisation time of flight (MALDI-TOF)/TOF analysis.

#### Central metabolic enzymes and the proteins involved in fermentative pathways appear more abundant at lower pH

*C. butyricum* CWBI 1009 utilises the Embden-Meyerhof-Parnas pathway for the conversion of glucose to phosphoenolopyruvate (PEP, Figure 5). The core metabolic proteins, such as glucose kinase (+2.52-fold more abundant at pH 5.2), phosphofructokinase 1 (+2.21), phosphopyruvate hydratase (+2.02) and pyruvate kinase (+1.88), that predominantly determine the carbon and electron flow from the carbohydrate substrate to the end-products were more abundant at pH 5.2 compared to pH 7.3 (Additional file 5: Table S11). Electrons derived from the main glycolytic nodes, namely pyruvate-ferredoxin oxidoreductase (PFOR) and to a lesser extent NADH-ferredoxin oxidoreductase (NFOR), are passed on by electron acceptors to the hydrogenases, thereby enabling the reversible reduction of the protons accumulated during the fermentation process to molecular hydrogen [28]. Also, the reducing equivalents necessary for N_2_ fixation are mainly obtained by the nitrogenase via reduced ferredoxin, which can be generated by the action of PFOR [29]. In line with these reports, seven redundant proteins identified as PFOR were all more abundant at pH 5.2 (on average + 2.37). By contrast, the relative abundance of type I glyceraldehyde-3-phosphate dehydrogenase, a tetrameric NAD-binding protein, was decreased at lower pH (on average -1.74), suggesting a limited flow of electrons via the NFOR node and towards the putative bifurcating [FeFe] hydrogenase (Hyd A8) [12,30]. Electron transferring flavoprotein (+1.73) and flavodoxin (+1.69) were also more abundant at lower pH, which was consistent with the RNA-seq data for the low pH condition.

Among the other proteins that were more abundant at acidic pH, a cysteine desulphurase NifS (+4.89) and an iron-sulphur cluster-binding protein (+1.92) were identified (Additional file 5: Table S11). The function of the former is to mobilise sulphur atoms for the biosynthesis of iron-sulphur (FeS) clusters. Both proteins are involved in the maturation process of various FeS proteins, such as hydrogenases and nitrogenases [31].

As regards the nitrogen metabolism, the 2D-DIGE analysis did not confirm the differential abundance of the nitrogenase, but did however show that glutamine synthetase was more abundant (by an average factor of +1.72) at lower pH. The mRNA level for the corresponding gene was also higher at the low-pH stage of the fermentation (Additional file 2: Table S4 and Additional file 3: Table S5). In free-living diazotrophs fixed N_2_ is assimilated by the organism in the form of ammonium to produce glutamine and glutamate. This occurs via the glutamine synthetase/glutamate synthase pathway in accordance with the equation: glutamate + ATP + NH_3_↔ glutamine + ADP + Pi [32]. Therefore, the ability to synthesise glutamate and glutamine is essential to the cellular metabolism, since they are involved in the incorporation of inorganic nitrogen into cell material; that is, the synthesis of new proteins [32,33]. Further ammonia incorporation occurs through the action of NAD(P)-specific glutamate dehydrogenase, and this protein also was found to be 1.81-fold more abundant at lower pH. Moreover, amino acids such as glutamate have been described as known osmoprotectants and have been shown to play a role in acid tolerance in, for example, *C. acetobutylicum* [15].

The HPLC analyses for metabolites indicated that, in addition to formate, acetate and butyrate were the main fermentation end-products obtained when *C. butyricum* CWBI 1009 was cultured under unregulated-pH conditions (Figure 1G). Therefore, it was not surprising that acetate and butyrate kinases were respectively 1.49- and 1.68-fold more abundant at the lower pH compared to pH 7.3. Additionally, acetyl-CoA acetyltransferase (thiolase), which catalyses the first steps of the butyrate synthesis pathway [32], was also more abundant at the lower pH (+1.51). Of the other core fermentative enzymes, type II acetaldehyde/alcohol dehydrogenase was also more abundant, suggesting that increased alcohol production was associated with decreasing pH.

### H_2_ production from glucose at fixed pH 5.2 is likely to be hydrogenase- mediated

Maintaining the *C. butyricum* CWBI 1009 culture at fixed pH 5.2 (Figure 1F) led to a 50% increase in cumulative H_2_ production (in comparison to the unregulated-pH culture; Figure 1D). Furthermore, bacterial fermentations at fixed acidic pH are commonly used for efficient H_2_ production. This is why glucose fermentations were also performed at two different fixed pH values, namely pH 7.3 (control fermentation with best growth) and pH 5.2 (test fermentation with best H_2_ production). In addition to the description of the fermentative profiles characteristic for the two fixed pH values, RT-qPCR was used to study the gene expression levels for the [FeFe] hydrogenases and the nitrogenase.

**Figure 6:**
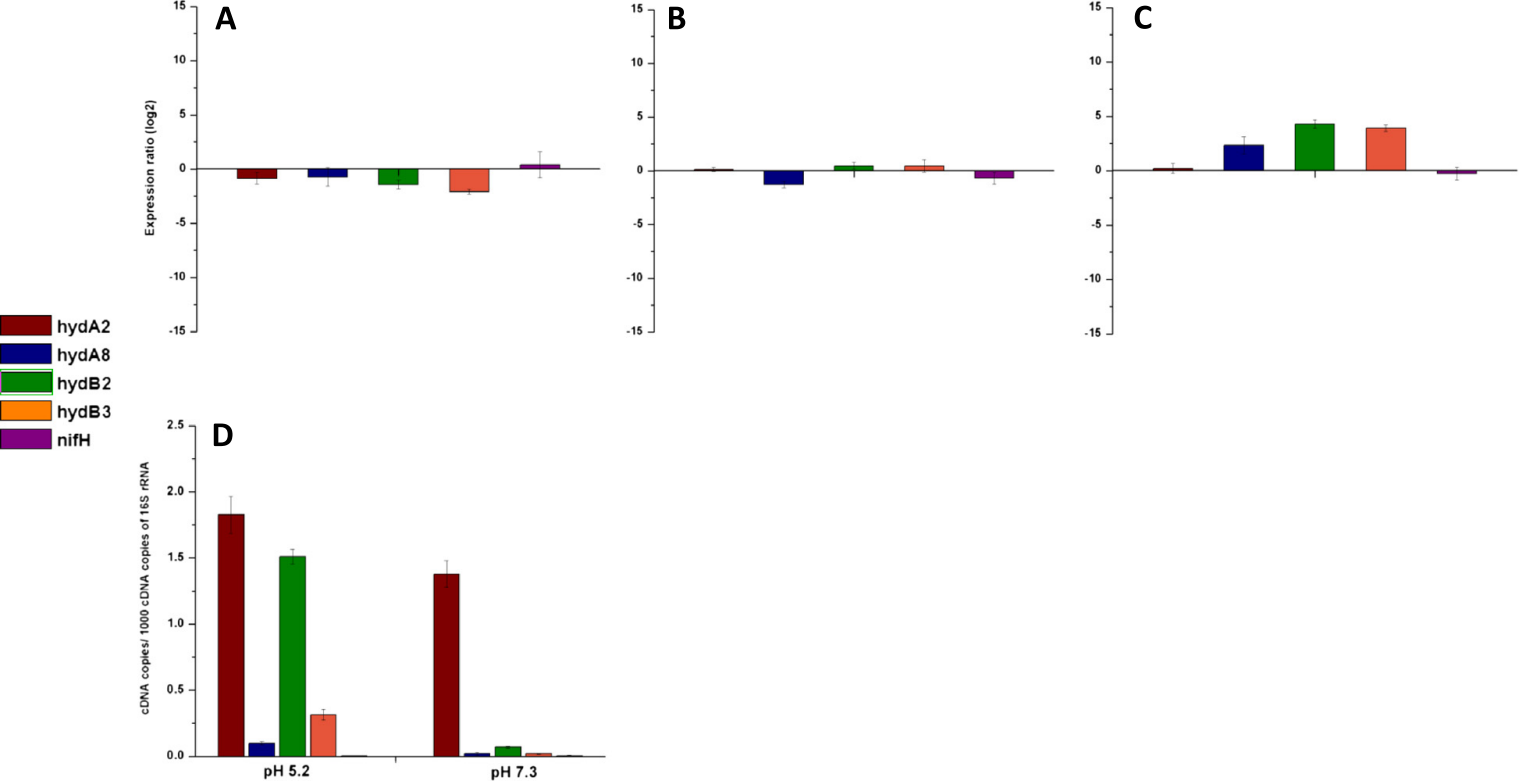
**Relative expression of [FeFe] hydrogenases and** ***nifH*** **genes determined by RT-qPCR for** ***Clostridium butyricum*** **CWBI 1009 during fixed pH 7.3 and 5.2 glucose fermentations. (A)** Fold change in the expression level of the *hydA2, hydA8, hydB2, hydB3, nifH* genes during fixed pH 7.3 glucose fermentation. **(B)** Fold change in the expression level of *hydA2, hydA8, hydB2, hydB3, nifH* genes during fixed pH 5.2 glucose fermentation. For **(A)** and **(B)** the values correspond to the late exponential (test sample) versus early exponential growth phase (control sample). **(C)** Fold change in the expression level of *hydA2, hydA8, hydB2, hydB3, nifH* genes during the fixed pH 5.2 versus 7.3 glucose fermentations. The values correspond to the late exponential growth stages from both experiments that correspond to the peak in H_2_ production; the culture at fixed pH 5.2 was used as a test sample, while pH 7.3 was a control sample. **(D)** Basal expression levels of the *hydA2, hydA8, hydB2, hydB3, nifH* gene transcripts during fixed-pH glucose fermentations. The expression level is shown as the number of cDNA copies per 1,000 cDNA copies of 16S rRNA. The values correspond to the late exponential growth stages from both experiments that correspond to the peak in H_2_ production.

At pH 7.3 no differential gene expression occurred throughout the fermentation (Figure 6A), as was expected given the barely detectable H_2_ production (Figure 1E). Surprisingly, at fixed pH 5.2, although the H_2_ production rate constantly increased during the fermentation, no change in the gene expression pattern was detected between the early and the late exponential stages of fermentation, neither for the nitrogenase nor for the [FeFe] hydrogenases (Figure 6B). This apparent lack of change in the temporal gene expression profile could be due to the fact that the bacterial pre-culture, used to inoculate the reactor, was pre-incubated at pH 5.2 as well. Therefore, the lower H_2_ production observed at the beginning of the fixed pH 5.2 fermentation (Figure 1F) was more likely attributable to low cell density rather than any differential [FeFe] hydrogenase gene expression. Since the pH was kept constant during the whole fermentation, the [FeFe] hydrogenases could not have been activated by a change in pH [34].

A direct comparison of the gene expression profiles for the same growth stage at the two different fixed pHs showed that the [FeFe] hydrogenase gene transcripts were indeed differentially abundant. During the late exponential growth phases (10 h incubation, Figure 1B and C), when the H_2_ production rate reached the maximum for both cultures (Figure 1E and F), the monomeric hydrogenase gene *hydA2* showed similar expression levels at fixed pH 5.2 and at fixed pH 7.3 (Figure 6C). Surprisingly, its basal expression at pH 5.2 was between 1.2- and 6 -fold higher than that for *hydB2* and for *hydB3* respectively, and as much as 18 times higher than that for the *hydA8* gene (Figure 6D). Hydrogenase A8 is a putative trimeric hydrogenase that requires both reduced ferredoxin and NADH to efficiently catalyse H_2_ production [30]. Its relatively low expression level compared to the other [FeFe] hydrogenases may suggest that there was less electron flow through the NFOR node than through the PFOR node (in line with the 2D-DIGE data). During the same late exponential growth phase the remaining two monomeric hydrogenase gene transcripts (*hydB2* and *hydB3*) were respectively 4.28- and 3.9-fold more abundant (*P* < 0.05, log_2_ scale) at fixed pH 5.2 compared to fixed pH 7.3.

The data obtained suggest two main conclusions. Firstly, based on the cDNA copy numbers, *hydA2* is the most abundant hydrogenase and seems to be insignificantly regulated under the two different fixed pH conditions. Secondly, both *hydB2* and *hydB3* are very strongly up-regulated at fixed pH 5.2 versus fixed pH 7.3; however, the basal expression level of *hydB2* at fixed pH 5.2 was around five times higher than that for *hydB3* (Figure 6D). Of particular interest also is the observation that during the unregulated-pH fermentation the basal expression levels of the four [FeFe] hydrogenase coding genes were lower than their expression levels at fixed pH 5.2 (10 h incubation, late exponential growth phase, Figure 4B and Figure 6D). For the two fixed-pH fermentations the expression level of the *nifH* gene was similar (Figure 6C), and was significantly lower than during the unregulated-pH glucose fermentation (Figure 4B). A Western blot analysis carried out on samples corresponding to the different fermentation stages for both fixed-pH cultures gave no positive signal for the NifH subunit (data not shown). This observation suggests that in contrast to the unregulated-pH glucose fermentation, where a strong up-regulation of nitrogenase genes was observed, H_2_ production at fixed pH 5.2 may be essentially hydrogenase-mediated.

To better understand the phenomenon of possibly differential nitrogenase- and hydrogenase-mediated H_2_ production in clostridia under different pH conditions, further investigation is required. Moreover, the genetic elements for the transcriptional control of the *nif* operon have not yet been well defined in clostridia [27]. Nevertheless, it is tempting to speculate about the significance of the differential carbon metabolisms (Figure 1G and I) observed for fermentation under unregulated- pH compared with fermentation under fixed pH 5.2 condition. This may be an indication of metabolic adaptations affecting the H_2_-producing enzymes, thereby leading to distinct H_2_ production profiles (Figure 1D and F). Interestingly, it has recently been shown that the nitrogenase-mediated H_2_ evolution in a photoheterotrophic bacterium *Rhodobacter sphaeroides* was enhanced by ethanol [35,36]. Oh *et al*. [36] reported a 60% increase in H_2_ production in the presence of ethanol in the ammonium-containing medium. Moreover, the authors showed that the increased nitrogenase activity was regulated at the level of *nifHDK* transcription and that ethanol was not used as a carbon source by the bacterium. Though the study discusses another type of bacterium, it can be compared with the unregulated-pH fermentation in our study, where both an increase in the production of ethanol and transcription of *nif* genes were detected and were concurrent with a higher H_2_ production. In contrast, during fixed pH 5.2 glucose fermentation, where no ethanol production was observed, the expression level of *nifH* was significantly lower as well.

Nevertheless, there are still unresolved questions concerning the metabolic fluxes and regulations in clostridia that need to be studied before a more grounded hypothesis can be made, especially as our study is the first to suggest, based on transcriptional analysis, a putative involvement of nitrogenase to the overall H_2_ production in this bacterial genus.

## Conclusions

This paper presents a biomolecular overview of the changes occurring when the metabolism of *C. butyricum* CWBI 1009 shifts to H_2_ production. The results show that the cellular metabolism for all the tested metabolites varied under all conditions and was pH dependent. The primary soluble metabolites were formate, acetate and butyrate, followed by ethanol and lactate. The highest H_2_ yield (1.78 ± 0.11 mol H_2_/mol glucose) was in accordance with previous studies with the same strain [9,10]. Further investigations into the genomic/proteomic changes associated with naturally decreasing pH and with H_2_ production indicated the differential regulation of numerous genes/proteins; some were known to be directly associated with H_2_ production (for example, hydrogenases and pyruvate-ferredoxin oxidoreductase). However the up-regulation of others, such as those involved in N_2_ assimilation, were more surprising, since H_2_ production in clostridia has never before been described as nitrogenase-mediated.

As the transcription levels of the [FeFe] hydrogenases and the nitrogenase coding genes varied significantly between different fermentations, transcriptional analyses indicated the need for further biochemical characterisation of the H_2_-producing enzymes, especially for the [FeFe] hydrogenases HydA8, B2 and B3, which have not yet been studied [6]. Additionally, the identification of multiple hypothetical genes/proteins (Additional file 2: Table S4) that were differentially regulated suggests that other unknown mechanisms may be governing H_2_ production in clostridia. This could therefore be fertile ground for future studies.

This work contributes to the field of dark fermentative hydrogen production by providing a multidisciplinary approach for the investigation of the processes involved in the molecular H_2_ metabolism of clostridia, which could in time lay the groundwork for further optimisation based on genetic engineering techniques [3].

## Methods

### Microorganism and growth conditions

The strain *Clostridium butyricum* CWBI 1009 was isolated from an anaerobic sludge and cultivated in a modified MDT medium as previously described [9]. The modified MDT medium contained, per litre of deionised water: glucose monohydrate (10 g), casein pepton (5 g), yeast extract (0.5 g), Na_2_HPO_4_ (5.1 g), KH_2_PO_4_ (1.2 g), MgSO_4_.7H_2_O (0.5 g), and cystein hydrochloride (0.5 g). The PCA (Plate Count Agar) medium, used to verify the absence of aerobic and facultative aerobic contaminants, contained per litre of deionised water: glucose monohydrate (1 g), casein peptone (5 g), yeast extract (2.5 g), and agar (15 g). All the chemicals used were of analytical or extra pure quality and were supplied by Merck, UCB and Sigma. Casein peptone and yeast extract were supplied by Organotechnie (La Courneuve, France).

### Reactor setup and experimental procedure

Fermentations were carried out in a 20-L laboratory-scale bioreactor (Biolafitte Niort, F) consisting of a double envelope and a stainless steel lid equipped with a butyl septum, 0.20-μm gas filters (Midisart, Sartorius) and tubing (for gas inlet, gas outlet and medium removal). Prior to inoculation the bioreactor and the medium were sterilised at 120°C for 20 min. Glucose and cystein were autoclaved separately in 2.5-L flasks to prevent Maillard reactions, and were then added sterilely to the tank before being cooled and purged with N_2_ or Ar. After inoculation with 1.7 L of pre-culture (obtained in 2-L hermetic bottles incubated for 24 hours at 30°C), the bioreactor had a final working volume of 17 L. Finally the pH was adjusted to either 7.3 or 5.2 ± 0.1 via automatic addition of sterile 1.5 N KOH combined with a Mettler Toledo probe (465-35-SC-P-K9/320) and needles inserted through the septum. For the unregulated-pH fermentation, after setting the initial pH value at 7.6 ± 0.1, the controller for base addition was turned off. Three biological replicates corresponding to each pH condition (unregulated pH, fixed pH 7.3 and fixed pH 5.2) were prepared and monitored for 25 hours. Throughout the fermentation, the bioreactor was maintained at 30°C and stirred at 100 rpm.

### Biogas and metabolite monitoring

The biogas flow rate was measured by a MilliGasCounter-1 PMMA Ritter flow meter connected to a computer (Rigamo V1.30-K1 software, acquisition every 30 seconds). The biogas production was also regularly checked with a second drum-type flow meter (Ritter TG01) connected in series. The proportion of hydrogen gas was determined using a gas chromatograph (GC) (Hewlett-Packard 5890 Series II) fitted with a thermal conductivity detector (TCD) and a 30 m × 0.32 mm GasPro GSC capillary column (Altech) in series with a 20 m × 0.25 mm CarboPLOT P7 column (Chrompak). The temperatures of the injections, the TCD chambers and the oven were maintained at 90°C, 110°C and 55°C, respectively. Nitrogen was used as the carrier gas in the column at a flow rate of 20 ml min^-1^. The harvested liquid samples were centrifuged at 13,000 g for 1 min, and the supernatant was filtered through a 0.2-μm cellulose acetate membrane (Minisart, Sartorius) before HPLC analysis for glucose, ethanol, lactate, acetate, formate and butyrate. HPLC was carried out using an Agilent 1110 series chromatograph (HP Chemstation software) with a Supelcogel C-610H column preceded by a Supelguard H pre-column (oven temperature 40°C). 0.1% H_3_PO_4_ (in Milli-Q water) was used for the isocratic mobile phase (flow rate of 0.5 ml min^-1^) using a differential refraction index detector (RID, heated at 35°C). The method lasted for 35 min at a maximum pressure of 60 bar. The data for the glucose and metabolite concentrations were used to calculate the mass balance (MB) of the glucose conversion as described previously [37].

### RNA extraction

For RNA extraction, the RiboPure™ -Bacteria (Ambion) extraction kit was used. The cells in a 2-ml suspension were harvested by centrifugation (16,000 g for 1 min), frozen in liquid nitrogen and stored at -80°C. The RNA extraction was performed according to the instruction manual. The total RNA was eluted by the addition of 50 μl of the preheated elution solution. Before reverse transcription, any contaminating DNA was removed by a double treatment with TURBO DNase (TURBO DNA-free™, Ambion), according to the instruction manual. In each step, the reaction mixture was incubated at 37°C for 30 min. After the second incubation step, the DNase was inactivated by the addition of the DNase inactivation reagent at a concentration of 20% of the volume of the treated RNA. The mix was then incubated for 2 min at room temperature and subsequently centrifuged for 1 min at 10,000 g to pellet the inactivation reagent. The absence of the genomic DNA contamination was confirmed by qPCR directly using 5 ng of total RNA. Total RNA concentrations were determined with a NanoVue spectrophotometer (GE Healthcare), and the RNA integrity was checked on a formaldehyde-agarose gel. Before RNA-seq, the RNA quality was checked on an automated electrophoresis system (Experion, BioRad). The RNA was stored at -80°C before analysis. First-strand cDNA was synthesised with the Reverse Transcription System (Promega, Madison, WI, USA) according to the manufacturer instructions. The total volume of the reaction mixture was 20 μl and contained 500 ng of the total RNA and 500 ng of the control mouse RNA (Quantum RNA™ ß-actin Internal Standards, Ambion). Control mouse RNA was used as an external standard. The reaction was primed with the random primers supplied with the kit. Obtained cDNAs were diluted in diethylpyrocarbonate (DEPC)-treated water to a final concentration of 5 ng initial total RNA/μl.

### mRNA sample preparation and RNA-seq analysis

10 μg of extracted total RNA was treated with the MICROB*Express*™ Kit (Ambion) to enrich for mRNA, by removing the 16S and 23S rRNA. Paired-end libraries were prepared according to the TruSeq™ RNA Sample Preparation Guide (Illumina). The library preparation and Illumina RNA sequencing was performed by the GIGA transcriptomics platform (Liège, Belgium). Obtained reads were aligned using the BWA software using default parameters [38]. Raw counts per gene were calculated based on the genome annotation of *Clostridium butyricum* CWBI 1009. Reads were allowed to map 50 bp upstream of the start codon or 50 bp downstream of the stop codon. Reads mapping to ribosomal or transporter RNA were removed from the raw counts data to prevent bias in detecting differential expression. Differential expression was calculated using the edgeR package (version 3.2.4) [39] in BioConductor (release 2.12, R version 2.15.0), resulting for each gene in a fold change and a corresponding *P*-value corrected for multiple testing. Genes with a 2-fold (log_2_) up or down-regulation and a corrected *P*-value lower than 0.05 were assigned as being differentially expressed.

### *Clostridium butyricum* CWBI 1009 genome sequencing

The *Clostridium butyricum* CWBI 1009 genome was sequenced by BaseClear (Leiden, The Netherlands) using the pair-end sequencing on the Illumina Hiseq 2000. Genome assembly was performed using Velvet version 1.2.10 [40], using a hash length of 29, a minimum contig length of 500 and a minimal coverage of 20. The Whole Genome Shotgun project was deposited at [DDBJ/EMBL/GenBank:ASPQ00000000]. The accession version described in this paper is version ASPQ01000000 (Additional file 1: Table S12).

### RT-qPCR

Species-specific primers and probes are listed in Table 1. ß-actin-specific primers were taken from the kit (QuantumRNA™ ß-actin Internal Standards, Ambion). The quantitative PCR amplifications were carried out with a Mini Opticon (BioRad). The DNA template used for a standard curve was prepared as previously described [41]. For the gene expression analysis, 1 μl of cDNA was used. The total volume of the PCR mix was 25 μl. Each reaction consisted of 1 × PCR mix (ABsolute™ Blue QPCR SYBR® Green Fluorescein Mix or ABsolute™Blue QPCR mix, Thermo Scientific), each primer and/or hydrolysis probe (HPLC cleaned, Biomers, Germany) at a final concentration of 150 nM. Each sample was analysed in triplicate. A ‘no template’ control was included in each run. The specificities of the primers were verified at the end of each qPCR reaction by performing the melting curve analysis (for a SYBR Green-based quantification). The standard curve preparation and the cycling conditions for SYBR Green chemistry were as previously described [41]. For the probe-based chemistry, the initial denaturation of 15 min was followed by 40 cycles of denaturation at 95°C for 15 s and primer annealing/amplification step at 60°C for 30 s. The reaction efficiency was calculated as factor specific [42] according to the equation: E = 10^-1/slope^. For gene expression analysis, the relative expression levels were calculated with a Relative Expression Software Tool 2009, REST© [43]. To estimate the up- and down-regulation of analysed genes, the obtained Cqs were compared to those of the reference genes and an external standard control gene. Two internal reference genes, 16S rRNA and *recA,* and one external RNA control gene (ß-actin) were used. An external mouse RNA was added to the sample RNA before the cDNA synthesis to correct for intrinsic and technical variations introduced throughout the experimental process [44]. The stability of the chosen reference genes and the integrity of each RNA sample were evaluated using the BestKeeper Excel-based tool [45] (data not shown).

**Table 1:** **List of** ***C. butyricum*** **CWBI 1009-specific primers and probes used in this study**

**Target gene**	**Primer/probe**	**Sequence 5’ → 3’**	**Length (bp)**	**Ref.**
*recA*	RecA-ButF	AAGCATTAGTGCGTTCTGGAG	97	[41]
	RecA-ButR	GAATCTCCCATTTCCCCTTC		
*hydA2*	ButA2F	ATAGTTGCAATGGCTCCTGC	250	This study
	ButA2R	TTTCTGCTTGCCTAACCCAT		
*hydA8*	ButA8F	TCTTTGGAGTTACAGGGGGA	188	This study
	ButA8R	TTCAGCATTTGCAAGACCAC		
*hydB2*	ButB2F	TGGTGGTGTATCAACTGCTG	168	This study
	ButB2R	TTGCATCCCATTCCTTCAAT		
*hydB3*	ButB3F	CAATGGTTGCTACAGGCAGA	168	This study
	ButB3R	CAAAAGCATCGAATAACGCA		
*16S rRNA*	16SButF	CCTGCCTCATAGAGGGGAAT	143	This study
	16SButR	GAGCCGTTACCTCACCAACT		
	HP16SBut^a^	CCGCATAAGATTGTAGTACCGCATGGTACA		
*nifH*	NifHButF	CATCAGCATTGGCTGAGATG	206	This study
	NifHButR	TGGTTCTGGTCCTCCTGATT		

^a^HP: hydrolysis probe.

### Western blot analysis

Whole cell protein extracts were prepared by sonicating the bacteria in TpW buffer (100 mM TRIS, 150 mM NaCl, 1mM EDTA). The cell lysates were centrifuged (16,000 g for 20 min) and protein concentrations in lysates were estimated using the Bradford assay, performed according to the manufacturer protocol (Fermentas). Western blot analysis was performed using a hen polyclonal affinity purified IgY raised against the NifH subunit of nitrogenase (Agrisera, Sweden). As a positive control, cell lysate of *Anabaena variabilis* was used. 5 μg of the whole protein extracts were directly subjected to 10% SDS-PAGE and then blotted onto Immobilon-P membrane (Millipore). Membranes were blocked for 1 h with TBST (20 mM Tris, 140 mM NaCl, 0.1% Tween, pH 7.6) containing 3% BSA, and then incubated with 1:2,000 diluted antibody in TBST, overnight at 4°C. As secondary antibody, a 1:10,000 dilution of rabbit anti-chicken IgG coupled to alkaline phosphatase (Sigma) was used. Reactive protein bands were detected using NBF/BdP reagents (Sigma).

### Sample preparation for 2D-DIGE

Freshly harvested cells were resuspended in a denaturation buffer (7 M urea, 2 M thiourea, 2% ASB-14, 20 mM DTT, Complete EDTA free [Roche], 1 mM EDTA pH 8.5, 50 mM Tris-HCl pH 7.5) and intensively vortexed for 30 min at room temperature. Subsequently, the suspensions were briefly sonicated and centrifuged at 10,000 g for 10 min to remove any insoluble material. In order to discard the remaining salts, fatty acids and nucleic acids, the protein extracts were precipitated three times and cleaned twice with a 2D-clean up Kit (GE Healthcare). Protein pellets were then resuspended in a DIGE labelling buffer (7 M urea, 2 M thiourea, 2% ASB-14, EDTA free anti-protease cocktail [Roche], 0.5 mM EDTA 50 mM TRIS adjusted at pH 8.5). The protein concentration was estimated with an RC/DC protein assay kit (BioRad Laboratories), and was adjusted to a value between 5 and 10 mg/ml for optimal CyDye labelling. Each sample (containing 25 μg of protein) was labelled with 0.2 nmol of either Cy3 or Cy5 (minimal labelling). At the same time, an internal standard consisting of equimolar amounts of the two samples was labelled with Cy2 (GE Healthcare). The labelling reaction was stopped after 30 min by adding 5 nM of lysine. The Cy2, Cy3- and Cy5-labelled proteins were pooled together prior to isoelectrofocussing with the IPGphor3 IEF System (GE Healthcare). Pooled samples were reduced by adding 20 mM DTT, resuspended in a Drystrip rehydration buffer (7 M urea, 2 M thiourea, 2% ASB-14 w/v, 0.6% IPG Buffer [GE Healthcare] v/v), and then supplemented with Destreak solution (GE Healthcare), to provide a final volume of 450 μl which was spread on a 24-cm regular strip holder. The 3-11 NL IPG Drystrips (GE Healthcare) were passively rehydrated in the strip holder for 10 h at 20°C prior to running the IEF under the following conditions: 50 V for 2 h (step), 200 V for 200 Vh (step), 500 V for 150 Vh (gradient), 500 V for 500 Vh (step), 1,000 V for 500 Vh (gradient), 8,000 V for 13,500 Vh (gradient), 8,000V for 8,0000 Vh (step) and 500 V for 10 h (step); with a maximum current setting fixed at 50 μA [46]. After the first electrophoretic migration (first dimension), the strips were reduced in an equilibration buffer (50% glycerol v/v, 4% SDS w/v, 6 M urea, 50 mM Tris-HCl adjusted to pH 8.8) and incubated with 300 mM DTT for 15 min. The strips were then alkylated for 15 min in the same equilibration solution with DTT replaced by 350 mM of iodoacetamide. After equilibration, the strips were put on top of a 12.5% acrylamide gel in a Laemmli SDS electrophoresis buffer (25 mM Tris, 192 mM glycine, 1% SDS w/v). Electrophoresis was carried out overnight at 1 W/gel. Following migration, the gels were scanned (Typhoon 9400, GE Healthcare) at a resolution of 100 μm for the excitation wavelengths corresponding to each CyDye. The scanner generated 18 gel images for each biological replicate (9 images for the comparison between the two pH values and 6 images for the internal standard) which were analysed with the DeCyder V7.0 software (GE Healthcare). The co-detection of the three CyDye-labeled forms for each spot was done using the Differential In-gel Analysis (DIA) module. Statistical analysis was carried out in the Biological Variation Analysis (BVA) module after inter-gel matching. Protein spots that resulted in a statistically significant Student’s *t*-test value (*P* < 0.05, n = 6) were considered as being differentially abundant at pH 5.2 compared to pH 7.3.

### In-gel digestion and mass spectrometry

Protein identification was performed using preparative gels (150 μg of loaded material) prepared under the same experimental conditions, but with only one CyDye to label the protein sample (Cy5). In addition, the gel plates were treated with a Bind-silane solution for spot picking. The resulting scanned gels were matched with the BVA module. Matched spots presenting a statistical difference were detected using the Ettan Dalt Spot Picker (GE Healthcare). Subsequently, the proteins in the gel pieces were washed (three times with Milli-Q water and 100% ACN and three times with 25 mM NH_4_HCO_3_ and 100% ACN) to remove excess detergent and buffer. After a final dehydration step in ACN, the gel pieces were rehydrated and treated with 2 μl of a 5 ng/μl trypsin proteomic grade solution (Roche) for 2 h at 4°C, to ensure sufficient diffusion in the gel. The temperature was then raised to 37°C for an overnight digestion. After tryptic digestion, the resulting peptides were extracted from the gel pieces by adding 5 μl of a 1% trifluoroacetic acid (TFA) v/v, 30% ACN v/v solution, and vortexing for 30 min. 1 μl of the resulting extract was dropped on a 384-600 MTP Anchorship MALDI target plate (Bruker Daltonic), previously spotted with a 3% w/v HCCA matrix (Sigma) dissolved in acetone. Each drop was washed 3 times with a 10 mM (NH_4_)_2_(HPO_4_) solution. Protein identification was carried out with MALDI-TOF/TOF instrumentation (Ultraflex II, Bruker Daltonic) in MS and MS/MS modes and the Mascot search engine was configured with a maximal mass error rate at 100 ppm [47]. Protein identification was performed with the Biotools software (Bruker) using the Mascot search engine on the *Clostridium butyricum* 5521 protein database (Additional file 5: Table S10).

## Additional files

Additional file 1: Figure S1. Double-log scatter of sequence reads and the coefficient of determination (R^2^) for the biological replicates of the RNA-seq reads mapped to the genome of *Clostridium butyricum* CWBI 1009. **Figure S2.** Correlation of RNA-seq data with RT-qPCR for *Clostridium butyricum* CWBI 1009 cultivated in a 20L batch bioreactor with glucose (10 g/L) under unregulated-pH conditions. **Figure S3.** 2D-gel pattern of the *Clostridium butyricum* CWBI 1009 proteome. **Table S1.** Bioreactor performance of *Clostridium butyricum* CWBI 1009 cultivated in a 20L batch bioreactor with glucose (10 g/L) under unregulated-pH conditions. **Table S2.** Bioreactor performance of *Clostridium butyricum* CWBI 1009 cultivated in a 20L batch bioreactor with glucose (5 g/L) at fixed pH 7.3 and 5.2. **Table S3.** Summary of *Clostridium butyricum* CWBI 1009 RNA-seq data results. **Table S12.** Summary of *Clostridium butyricum* CWBI 1009 genome information. Additional file 2:
**RNA-seq data. Table S4.** List of the most highly expressed genes at pH 6.3 (column indicated in bold type) during glucose fermentation under unregulated-pH conditions. Additional file 3:
**RNA-seq data. Table S5.** Differentially regulated genes in *C. butyricum* CWBI 1009 in response to the decline in pH (unregulated-pH glucose fermentation) as analysed by RNA-seq. Additional file 4:
**RNA-seq data.** **Table S6.** Acid stress response: alkalisation of the internal/external environment. Genes and pathways regulated at acidic pH 6.3 versus 7.3 during glucose fermentation under unregulated-pH conditions (RNA-seq data). **Table S7.** Acid stress response: cellular transport. Genes differentially regulated at acidic pH 6.3 versus 7.3 during glucose fermentation under unregulated-pH conditions (RNA-seq data). **Table S8.** Acid stress response: transcriptional regulators and other regulatory proteins. Genes differentially regulated at acidic pH 6.3 versus 7.3 during glucose fermentation under unregulated-pH conditions (RNA-seq data). **Table S9.** Acid stress response: activation of sporulation. Genes differentially regulated at acidic pH 6.3 versus 7.3 during glucose fermentation under unregulated-pH conditions (RNA-seq data). Additional file 5:
**2D-DIGE data. Table S10.** Protein identification table generated with the Biotools software (Bruker) using the Mascot search engine on the *Clostridium butyricum* 5521 protein database. **Table S11.** Proteins identified as differentially abundant in *C. butyricum* CWBI 1009 in response to the decline in pH during unregulated-pH glucose fermentation (2D-DIGE analysis). Additional file 6:
**Argon discussion. Figure S4.** Characteristics of the glucose fermentation for *Clostridium butyricum* CWBI 1009 performed under unregulated-pH conditions and Ar atmosphere. **Figure S5.** Relative expression of [FeFe] hydrogenases and *nifH* genes determined by RT-qPCR. Western blot analysis for NifH subunit of *Clostridium butyricum* CWBI 1009 during unregulated-pH glucose fermentation under N_2_ and Ar atmospheres. **Table S13.** Bioreactor performance of *Clostridium butyricum* CWBI 1009 cultivated in a 20L batch bioreactor with glucose (10 g/L) under unregulated-pH conditions and N_2_ or Ar atmospheres.

## Abbreviations

2D-DIGE: Two-dimensional difference in gel electrophoresis
CDS: Coding DNA sequence
NFOR: NADH-ferredoxin oxidoreductase
PEP: Phosphoenolopyruvate
PFOR: Pyruvate-ferredoxin oxidoreductase
RPKM: Reads per kilobase per million mapped reads
RT-qPCR: Reverse transcription quantitative real-time PCR
