# Supplementary material for: Genome-wide transcriptional analysis suggests hydrogenase- and nitrogenase-mediated hydrogen production in Clostridium butyricum CWBI 1009
Source: Biotechnol Biofuels. 2015 Feb 22;8:27. doi: 10.1186/s13068-015-0203-5 (PMC4342158; doi:10.1186/s13068-015-0203-5)
Supplement: Additional file 1: Figure S1. — Double-log scatter of sequence reads and the coefficient of determination (R2) for the biological replicates of the RNA-seq reads mapped to the genome of Clostridium butyricum CWBI 1009. Figure S2. Correlation of RNA-seq data with RT-qPCR for Clostridium butyricum CWBI 1009 cultivated in a 20L batch bioreactor with glucose (10 g/L) under unregulated-pH conditions. Figure S3. 2D-gel pattern of the Clostridium butyricum CWBI 1009 proteome. Table S1. Bioreactor performance of Clostridium butyricum CWBI 1009 cultivated in a 20L batch bioreactor with glucose (10 g/L) under unregulated-pH conditions. Table S2. Bioreactor performance of Clostridium butyricum CWBI 1009 cultivated in a 20L batch bioreactor with glucose (5 g/L) at fixed pH 7.3 and 5.2. Table S3. Summary of Clostridium butyricum CWBI 1009 RNA-seq data results. Table S12. Summary of Clostridium butyricum CWBI 1009 genome information. [file 13068_2015_203_MOESM1_ESM.docx]

**Additional file 1**

**B**

**A**


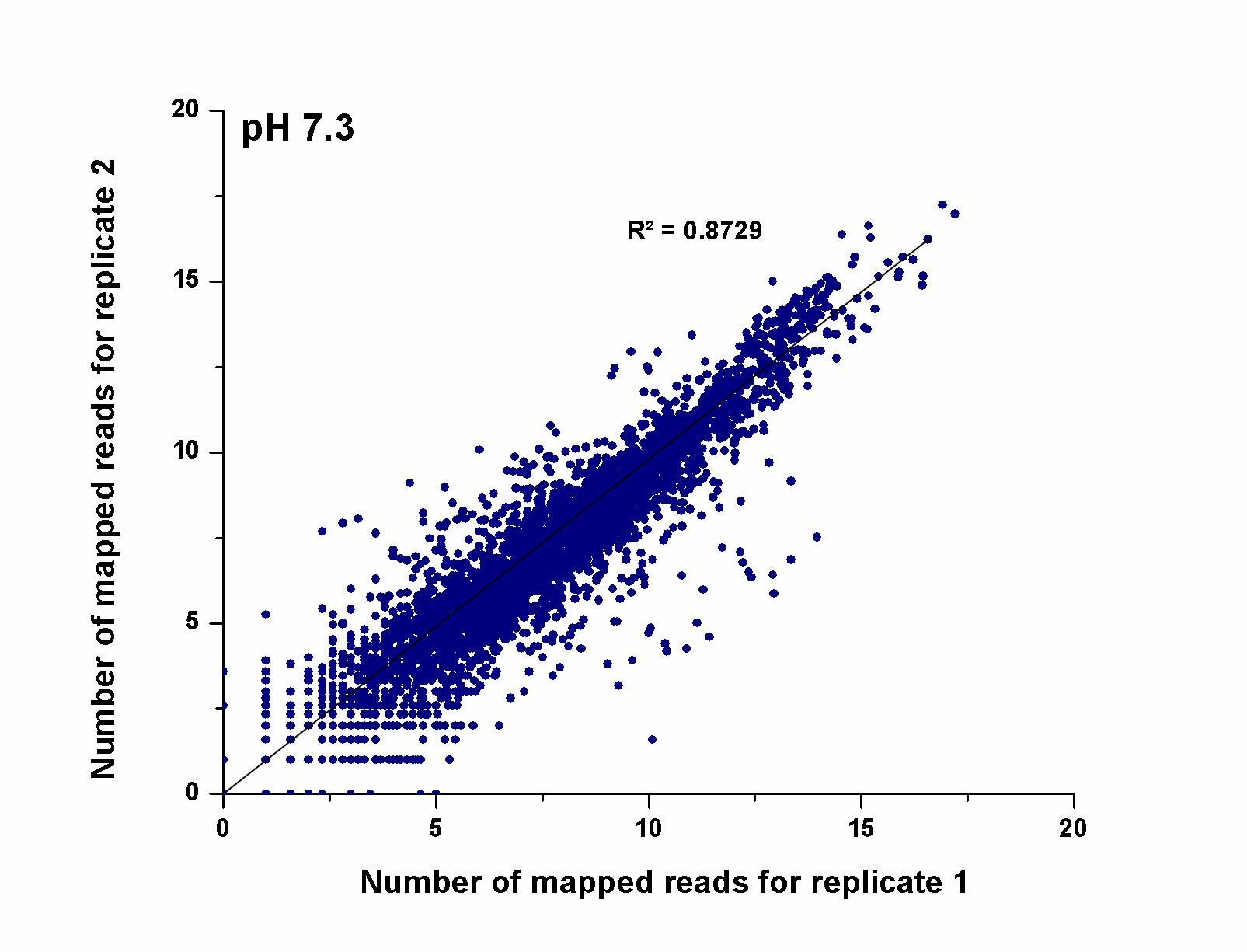

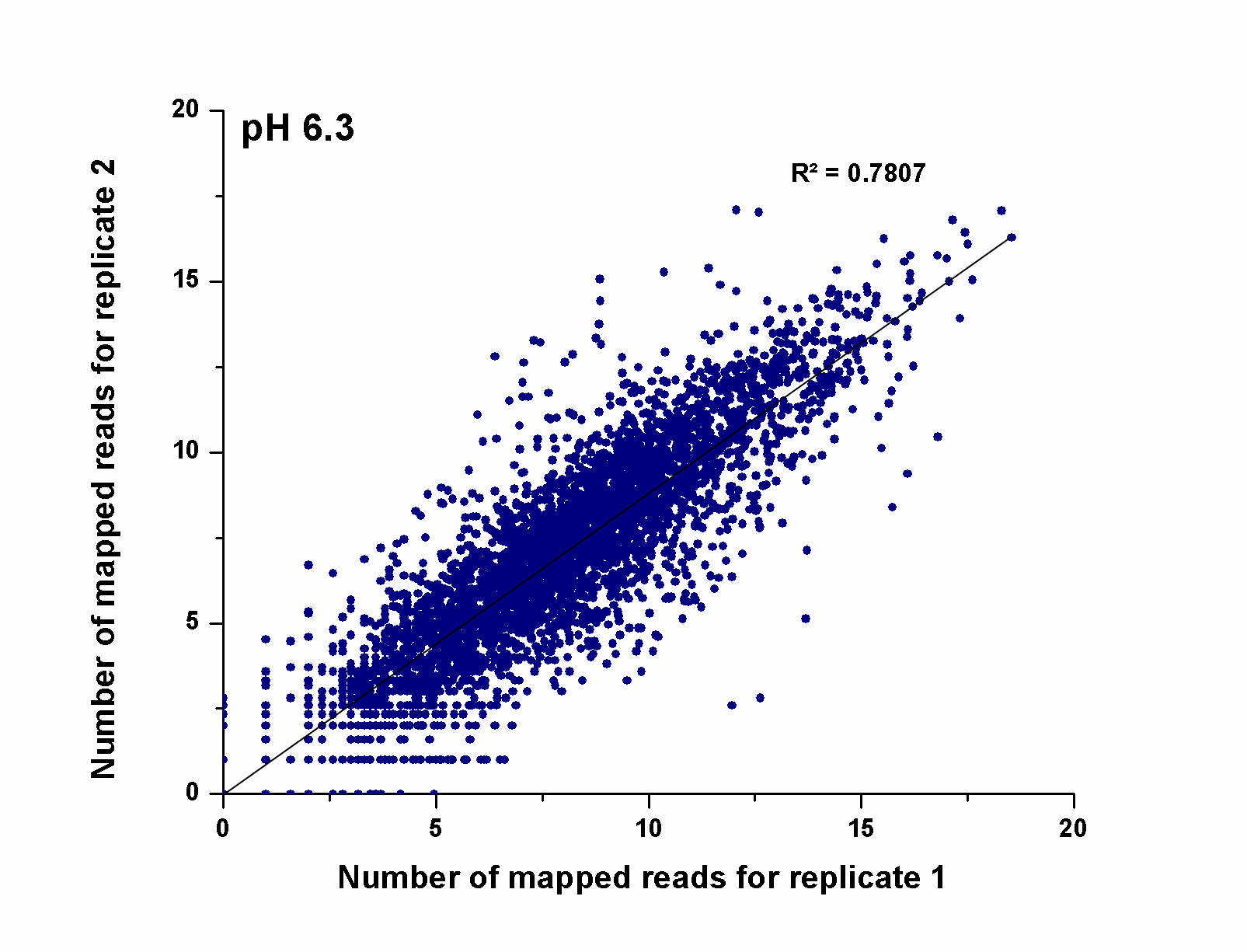


**Figure S1. Double-log scatter of sequence reads and the coefficient of determination (R^2^) for the biological replicates of the RNA-seq reads mapped to the genome of *Clostridium butyricum* CWBI 1009.**

Reproducibility between biological replicates for samples corresponding to pH values 7.3 **(A)** and pH 6.3 **(B)**. The reads from two independent libraries, excluding reads that mapped to rRNA, reads that did not map uniquely to the genome and reads that did not map at all to the genome, were mapped to the genome of *C. butyricum* CWBI 1009. The number of reads between the two libraries was normalized and the absolute number of reads that mapped to each CDS was compared.


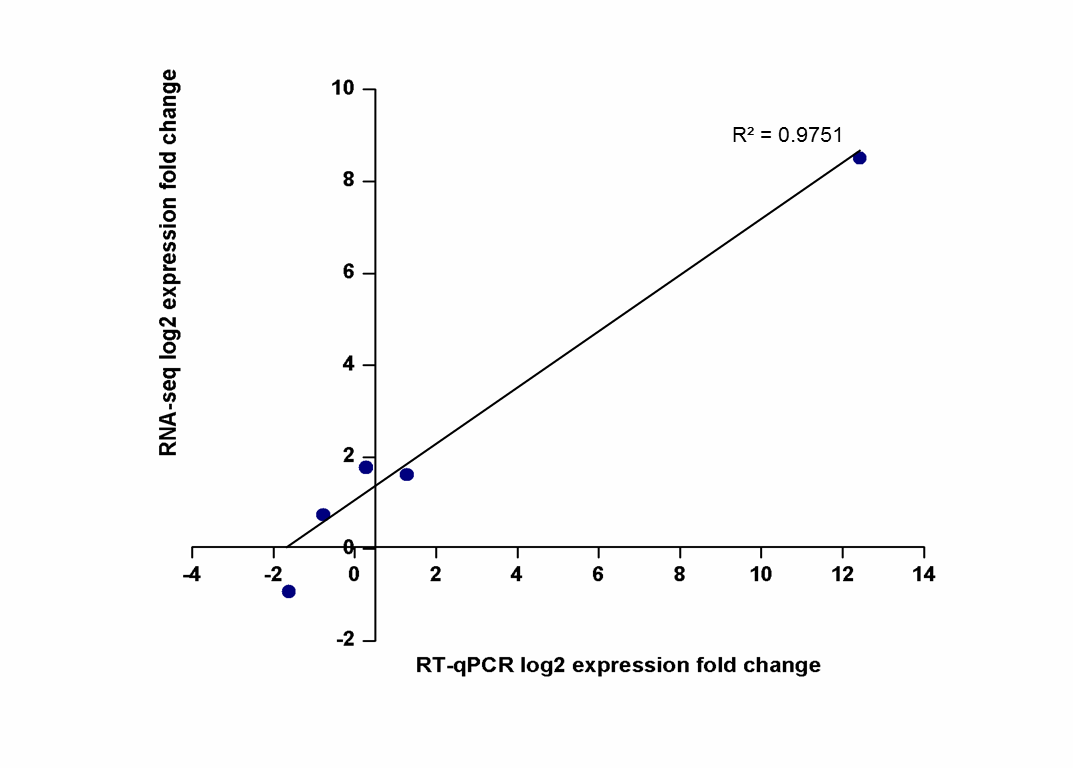


**Figure S2. Correlation of RNA-seq data with RT-qPCR (analysed genes *hydA2, hydA8, hydB2, hydB3* and *nifH*) for *Clostridium butyricum* CWBI 1009 cultivated in a 20 L batch bioreactor with glucose (10 g/ L) under unregulated-pH conditions.**

**
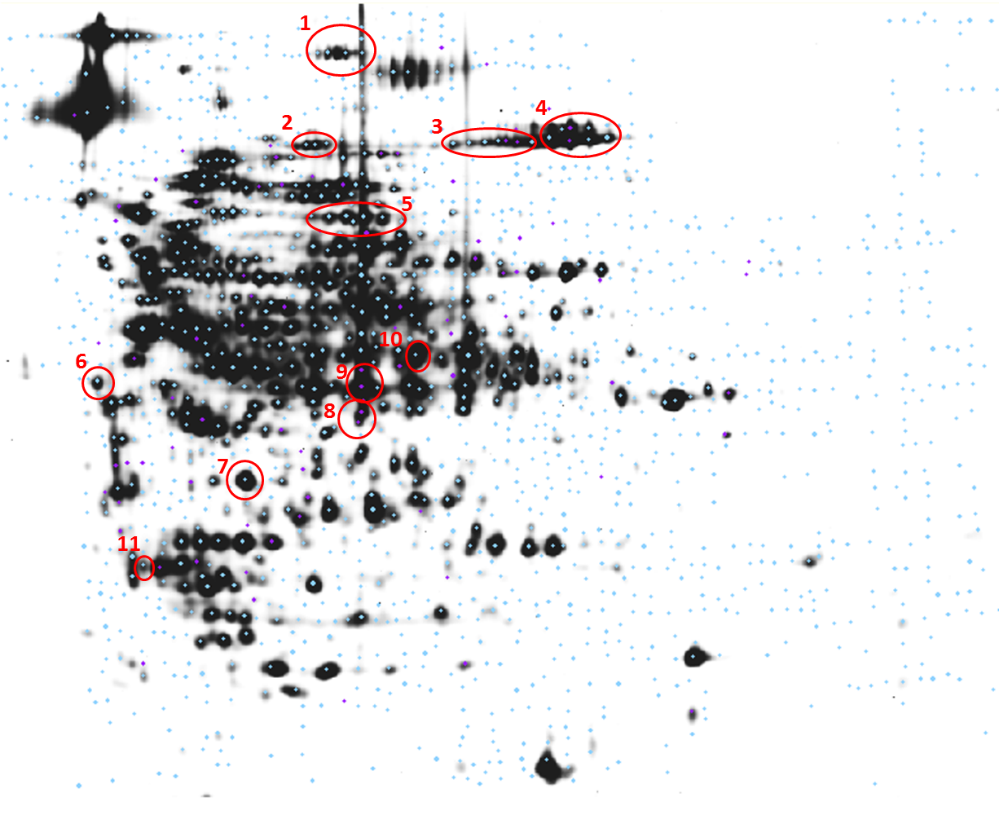
**

**Figure S3. 2D-gel pattern of the *Clostridium butyricum* CWBI 1009 proteome.**

The gel pattern displayed here is the reference gel (master gel). Some important regulated proteins are encircled: 1) pyruvate:ferredoxin oxidoreductase; 2) pyruvate kinase; 3) aldehyde-alcohol dehydrogenase 2; 4) phosphopyruvate hydratase; 5) formate C-acetyltransferase isoform; 6) glucose kinase; 7) acetate kinase; 8) flavodoxin; 9) butyrate kinase; 10) NAD(P)-specific glutamate dehydrogenase; 11) acetyltransferase.

**Table S1. Bioreactor performance of *Clostridium butyricum* CWBI 1009 cultivated in a 20 L batch bioreactor with glucose (10 g/ L) under unregulated-pH conditions.**

|  | **Growth** | **Growth rate** | **Glucose uptake** | **Biogas** | **H_2_ content^a^** | **H_2_** | **CO_2_** | **H_2_ yield** | **H_2_ rate** |
| --- | --- | --- | --- | --- | --- | --- | --- | --- | --- |
|  | DO_600nm_ | div.h^-1^ | g glucose/ h | L | % | L | L | mol H_2_/  mol glucose | L H_2_/ h |
|  | 2.5 ±0.1 | 0.50 ±0.02 | 0.84 ±0.06 | 17.16 ±0.84 | 63 ±4 | 10.80 ±0.44 | 6.36 ±0.28 | 1.78 ±0.11 | 1.56 ±0.15 |

^a^The H_2_ content is the average of all the measurements carried out during the exponential growth phase (5-10 h of fermentation).

**Table S2. Bioreactor performance of *Clostridium butyricum* CWBI 1009 cultivated in a 20 L batch bioreactor with glucose (5 g/ L) at fixed pH 7.3 and 5.2.**

|  | **Growth** | **Growth rate** | **Glucose uptake** | **Biogas** | **H_2_ content^a^** | **H_2_** | **CO_2_** | **H_2_ yield** | **H_2_ rate** |
| --- | --- | --- | --- | --- | --- | --- | --- | --- | --- |
|  | OD_600nm_ | div.h^-1^ | g glucose/ h | L | % | L | L | mol H_2_/  mol glucose | L H_2_/ h |
| **pH 7.3** | | | | | | | | | |
|  | 3.03 ±0.03 | 0.45 ±0.05 | 0.96 ±0.08 | 2.90 ±0.23 | 48.05 ±1.49 | 1.41 ±0.15 | 1.50 ±0.08 | 0.23 ±0.02 | 0.21 ±0.03 |
| **pH 5.2** | | | | | | | | | |
|  | 2.26 ±0.007 | 0.27 ±0.02 | 0.44 ±0.03 | 23.32 ±1.01 | 64.8 ±1.13 | 14.93 ±0.65 | 8.41 ±0.36 | 1.95 ±0.09 | 1.11 ±0.06 |

^a^The H_2_ content is the average of all the measurements carried out during the exponential growth phase (after 5-10 h of fermentation).

**Table S3. Summary of *Clostridium butyricum* CWBI 1009 RNA-seq data results.**

|  | **Biological replicate 1** | | | **Biological replicate 2** | |
| --- | --- | --- | --- | --- | --- |
|  | **pH 7.3** | **pH 6.3** | **pH 5.2** | **pH 7.3** | **pH 6.3** |
| **Total number of reads** | 21 056 498 | 20 633 894 | 16 943 840 | 18 605 474 | 19 986 690 |
| **Number of reads mapped uniquely** | 17 518 025 | 16 650 950 | 13 790 868 | 15 191 014 | 16 313 068 |
| **Number of reads corresponding to rRNA (%)** | 12 422 537  (70.91%) | 11 617 345  (69.77%) | 12 041 006  (87.31%) | 9 776 799  (64.36%) | 6 842 463  (41.94%) |

**Table S12. Summary of *Clostridium butyricum* CWBI 1009 genome information.**

| **Genome size^a^** | 4 491 619 bp |
| --- | --- |
| **DNA coding region** | 3 719 539 bp |
| **% DNA coding region** | 82.81% |
| **Number of contigs** | 340 |
| **N50** | 26 861 |
| **max contig length** | 120 959 |
| **GC content** | 28.52% |
| **Total genes** | 3999 |

^a^The Whole Genome Shotgun project was deposited at DDBJ/EMBL/GenBank under the accession ASPQ00000000.

The version described in this paper is version ASPQ01000000.
