# Supplementary material for: Genome-wide transcriptional analysis suggests hydrogenase- and nitrogenase-mediated hydrogen production in Clostridium butyricum CWBI 1009
Source: Biotechnol Biofuels. 2015 Feb 22;8:27. doi: 10.1186/s13068-015-0203-5 (PMC4342158; doi:10.1186/s13068-015-0203-5)
Supplement: Additional file 6: — Argon discussion. Figure S4. Characteristics of the glucose fermentation for Clostridium butyricum CWBI 1009 performed under unregulated-pH conditions and Ar atmosphere. Figure S5. Relative expression of [FeFe] hydrogenases and nifH genes determined by RT-qPCR. Western blot analysis for NifH subunit of Clostridium butyricum CWBI 1009 during unregulated-pH glucose fermentation under N2 and Ar atmospheres. Table S13. Bioreactor performance of Clostridium butyricum CWBI 1009 cultivated in a 20L batch bioreactor with glucose (10 g/L) under unregulated-pH conditions and N2 or Ar atmospheres. [file 13068_2015_203_MOESM6_ESM.docx]

**Additional file 6**

**Discussion of the results for unregulated-pH glucose fermentation under an argon atmosphere**

Though [FeFe] hydrogenases are recognized as the main H_2_-producing enzymes in clostridia [1], our RNA-seq results suggested a nitrogenase-mediated H_2_ production in *C. butyricum* under N_2_ with unregulated-pH. Moreover, although nitrogenase is known to produce H_2_ as a by-product of N_2_ fixation, in the early eighties it was already reported that the enzyme can act as an ATP-powered hydrogenase and produce only H_2_ in the absence of N_2_ [2]. Therefore, to check if this was the case with clostridia, we cultured *C. butyricum* CWBI 1009 under unregulated-pH conditions in an N_2_-free atmosphere in which argon was used instead of nitrogen (indicated as Ar) to initiate the anaerobic conditions in the bioreactor. The pattern of the metabolites produced ([Fig. 1. G and Fig. S4. C](file:///D:\Temp\Fig.%201.docx)), the growth curves (Fig. 1. A and Fig. S4. A) and the cumulative productions of H_2_ ([Fig. 1. D and Fig. S4. B](file:///D:\Temp\Fig.%201.docx)) were similar under both conditions, suggesting that Ar had only a minor influence on the main fermentative pathways. However we observed that pH stress resulted in dissimilar H_2_ production profiles under N_2_ and Ar; a higher production rate occurred under N_2_ (1.56 ± 0.15 vs 0.93 L H_2_/ h), but with a much shorter production period (7.5 vs 20 h; [Fig. 1. D and Fig. S4. B](file:///D:\Temp\Fig.%201.docx)). Logically one would expect the main H_2_-producing enzymes to be differentially expressed as well. To confirm it we used RT-qPCR to analyse the expression levels for the three monomeric [FeFe] hydrogenase genes (*hydA2, hydB2* and *hydB3*), the trimeric [FeFe] hydrogenase (*hydA8*) gene and the *nifH* gene. Our results demonstrated that, in contrast to the fermentation under N_2_, under the Ar there was an induction of the expression of three [FeFe] hydrogenase genes (*hydA8, hydB2* and *hydB3)* when the pH dropped to 5.2. This also corresponded with the highest H_2_ production rate observed during the Ar fermentation experiment (after 9-10 h of fermentation, Fig. S4. B).

When comparing the respective stages of glucose fermentations under an Ar versus an N_2_ atmosphere, we observed that for the stages associated with peak H_2_ production, the expression level of *hydA8*, *hydB2* and *hydB3* was 0.78, 2.58 and 3.75 fold higher (log_2_ scale) respectively under Ar ([Fig. S5. A](file:///C:\Temp\Fig.%204.docx)). The expression level of the *hydA2* gene was unchanged between the two conditions. Hydrogenase HydA2 seems to be the only *C. butyricum* CWBI 1009 [FeFe] hydrogenase that is relatively constantly expressed under different pH and N_2_-availability conditions. The up-regulated hydrogenase HydB3 is similar in modular structure to the well characterized HydA2 [3], though neither its gene expression nor the protein itself have been studied before. Here we clearly demonstrated that [FeFe] hydrogenases other than HydA2 may also influence H_2_ production in clostridia. The most remarkable difference observed was that the average expression level of the *nifH* gene was more than 11-fold lower (log_2_ scale) during fermentation under Ar compared to N_2_ ([Fig. S5. A](file:///C:\Temp\Fig.%204.docx) and B), and that the NifH subunit could not be detected by Western blot analysis ([Fig. S5. C](file:///C:\Temp\Fig.%204.docx)). Interestingly, it has recently been reported that the presence of ethanol stimulated transcription of *nifHDK* genes by increasing the *nifA* expression, and enhanced the overall H_2_ production by 60% in *Rhodobacter sphaeroides* when grown in an ammonium-containing medium [4]. The metabolic similarities between *Rhodobacter* and clostridia may be limited, but the work of Oh *et al.* [4] illustrates the existence of complex regulation patterns that are still far from being deciphered. As shown in Figs. 1. G and S4. C, it is interesting to note that ethanol is indeed produced in both unregulated-pH fermentations under N_2_ and Ar. In our study ammonium must have been produced by nitrogenase activity under N_2_, but not when N_2_ was replaced with Ar. If there exists a similar regulation by ethanol when ammonium is present, its absence could potentially explain the lower transcriptional level of *nif* genes under Ar. However, a better understanding of the differences between the mechanisms leading to H_2_ production in clostridia under these two atmospheric conditions studied would require a more detailed analysis of the Ar sample at the transcriptomic or proteomic level (this was not performed as it was beyond the scope of this study). Additionally [FeFe] hydrogenase and nitrogenase activity assays could help to evaluate the contribution of each enzyme to the overall H_2_ production under different environmental conditions.

References

1. Vignais PM, Colbeau A: **Molecular biology of microbial hydrogenases.** *Curr Issues Mol Biol* 2004, **6:** 159-188.

2. Burgess BK, Wherland S, Newton WE, Stiefel EI: **Nitrogenase reactivity: insight into the nitrogen-fixing process through hydrogen-inhibition and HD-forming reactions.** *Biochem* 1981, **20:** 5140-5146.

3. Calusinska M, Happe T, Joris B, Wilmotte A: **The surprising diversity of clostridial hydrogenases: a comparative genomic perspective.** *Microbiol* 2010, **156:** 1575-1588.

4. Oh EK, Kim EJ, Hwang HJ, Tong X, Nam JM, Kim MS *et al*.: **The photoheterotrophic H_2_ evolution of *Rhodobacter sphaeroides* is enhanced in the presence of ethanol.** *Int J Hydrogen Energ* 2012, **37:** 15886-15892.

##
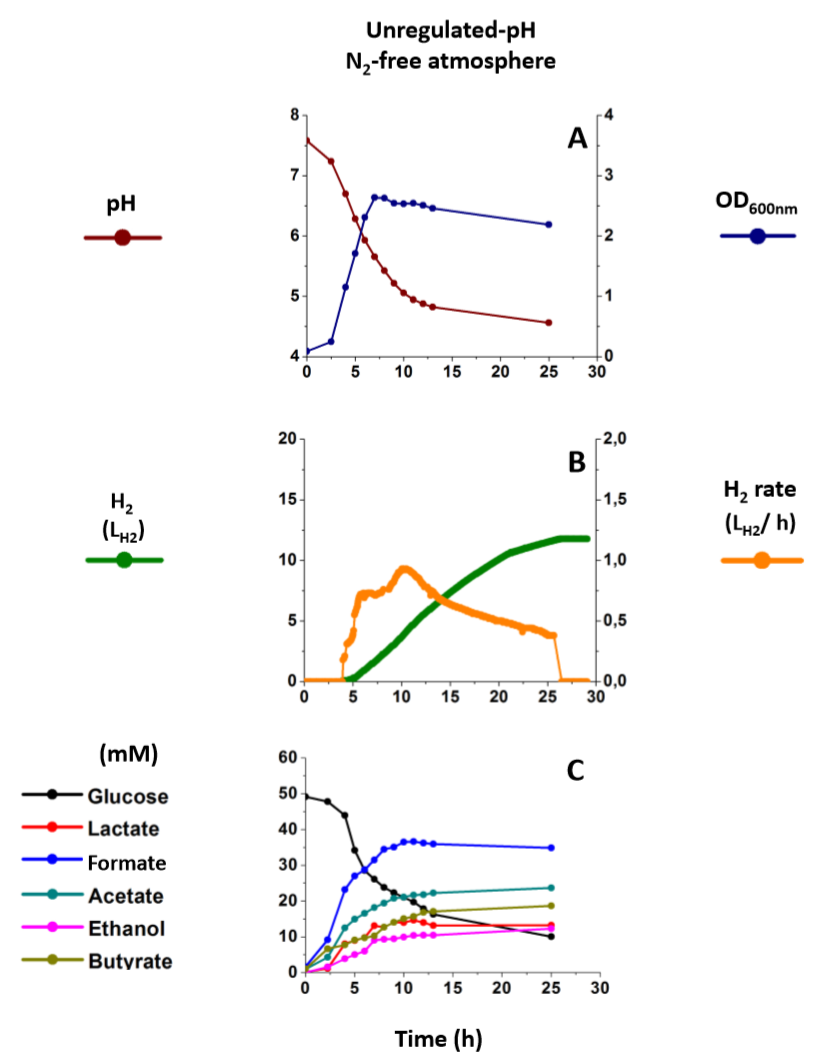


## Figure S4. Characteristics of the glucose fermentation for *Clostridium butyricum* CWBI 1009 performed under unregulated-pH conditions and Ar atmosphere.

(A) Growth curve (OD) and pH. (B) Hydrogen production rate (L/ h) and cumulative hydrogen production (L) profiles. (C) Glucose utilization and profiles of soluble metabolites (mM). The fermentation was performed in 20 L batch bioreactors with glucose as a substrate (10 g/ L).

**
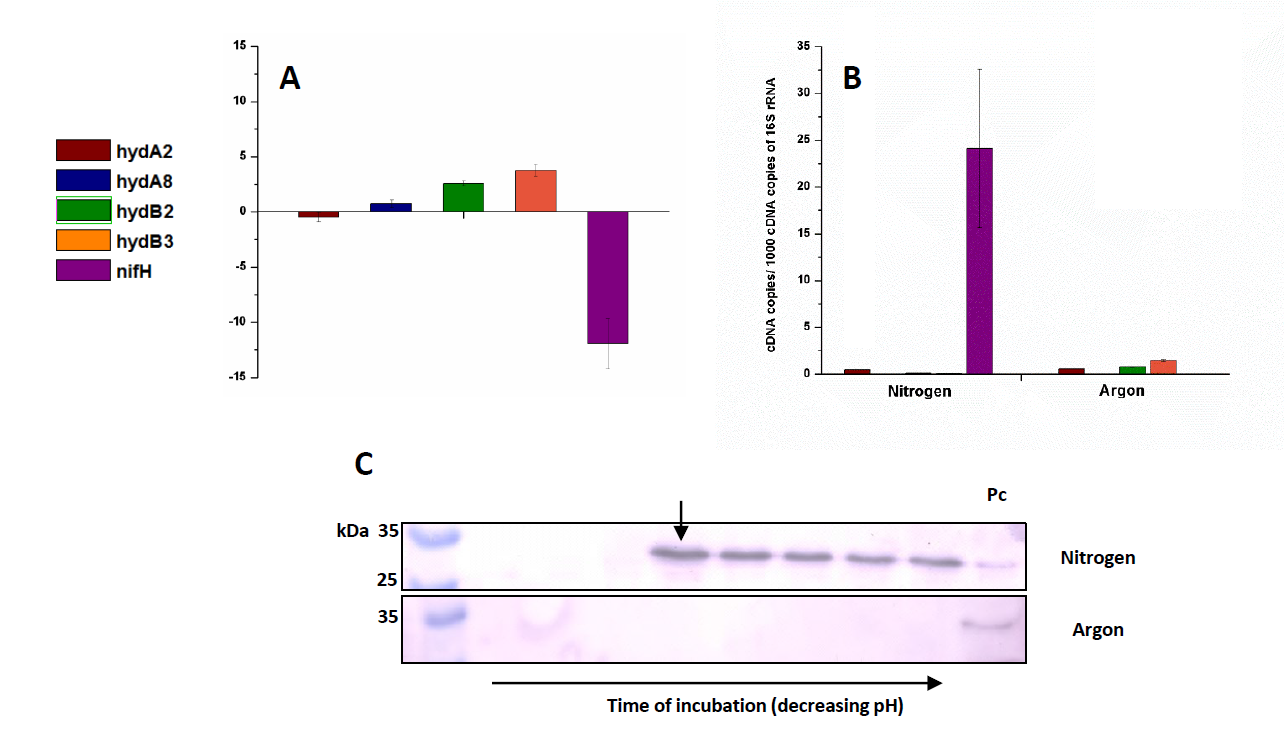
**

**Figure S5. Relative expression of [FeFe] hydrogenases and *nifH* genes determined by RT-qPCR for *Clostridium butyricum* CWBI 1009 during unregulated-pH glucose fermentation under N_2_ and Ar atmosphere. Western blot analysis for the NifH subunit.**

(A) Fold change in the expression level of *hydA2, hydA8, hydB2, hydB3, nifH* genes during unregulated-pH glucose fermentation under Ar (test sample) vs N_2_ (control sample). The values correspond to the late exponential growth stages from both experiments, characterized by the peak in H_2_ production and pH values of 5.2 for Ar and pH 6.3 for N_2_. (B) Basal expression levels of the *hydA2, hydA8, hydB2, hydB3, nifH* gene transcripts during unregulated-pH glucose fermentation under N_2_ and Ar. The expression level is shown as a number of cDNA copies per 1000 cDNA copies of 16S rRNA. The values correspond to the late exponential growth stages from both experiments corresponding to the peak in H_2_ production and pH values of 5.2 for Ar and pH 6.3 for N_2_. (C) Western blot analyses of the crude cellular extracts taken during unregulated-pH glucose fermentation under N_2_ and Ar. Time of incubation corresponds to the different growth stages starting from the beginning of the experiment until the pH dropped to a level of about 4.5 for both fermentations. The arrow indicates the pH 6.3. Pc-positive control.

**Table S13. Bioreactor performance of *Clostridium butyricum* CWBI 1009 cultivated in a 20 L batch bioreactor with glucose (10 g/ L) under unregulated-pH conditions and N_2_ or Ar atmosphere.**

|  | **Growth** | **Growth rate** | **Glucose uptake** | **Biogas** | **H_2_ content^a^** | **H_2_** | **CO_2_** | **H_2_ yield** | **H_2_ rate** |
| --- | --- | --- | --- | --- | --- | --- | --- | --- | --- |
|  | DO_600nm_ | div.h^-1^ | g glucose/ h | L | % | L | L | mol H_2_/  mol glucose | L H_2_/ h |
| **N_2_ atmosphere** | | | | | | | | | |
|  | 2.5 ±0.1 | 0.50 ±0.02 | 0.84 ±0.06 | 17.16 ±0.84 | 63 ±4 | 10.80 ±0.44 | 6.36 ±0.28 | 1.78 ±0.11 | 1.56 ±0.15 |
| **N_2_-free atmosphere** | | | | | | | | | |
|  | 2.6 | 0.59 | 0.77 | 19.97 | 58 | 11.78 | 8.19 | 1.39 | 0.93 |

^a^The H_2_ content is the average of all the measurements carried out during the exponential growth phase (5-10 h of fermentation).
